# Review of Natural Resources With Vasodilation: Traditional Medicinal Plants, Natural Products, and Their Mechanism and Clinical Efficacy

**DOI:** 10.3389/fphar.2021.627458

**Published:** 2021-04-01

**Authors:** Fei Tang, Hong-Ling Yan, Li-Xia Wang, Jin-Feng Xu, Cheng Peng, Hui Ao, Yu-Zhu Tan

**Affiliations:** ^1^ State Key Laboratory of Characteristic Chinese Medicine Resources in Southwest China, Pharmacy College, Chengdu University of Traditional Chinese Medicine, Chengdu, China; ^2^ Innovative Institute of Chinese Medicine and Pharmacy, Chengdu University of Traditional Chinese Medicine, Chengdu, China

**Keywords:** traditional medicinal plants, natural products, vasodilation, mechanism, cardiovascular and cerebrovascular diseases

## Abstract

For decades, chronic diseases including cardiovascular and cerebrovascular diseases (CCVDs) have plagued the world. Meanwhile, we have noticed a close association between CCVDs and vascular lesions, such as hypertension. More focus has been placed on TMPs and natural products with vasodilation and hypotension. TMPs with vasodilatory and hypotensive activities are mainly from *Compositae*, *Lamiaceae*, and *Orchidaceae* (such as *V. amygdalina Del.*, *T. procuinbens L.*, *M. glomerata Spreng.*, *K. galanga L.*, etc.) whereas natural products eliciting vasorelaxant potentials were primarily from flavonoids, phenolic acids and alkaloids (such as apigenin, puerarin, curcumin, sinomenine, etc.). Furthermore, the data analysis showed that the vasodilatory function of TMPs was mainly concerned with the activation of eNOS, while the natural products were primarily correlated with the blockage of calcium channel. Thus, TMPs will be used as alternative drugs and nutritional supplements, while natural products will be considered as potential therapies for CCVDs in the future. This study provides comprehensive and valuable references for the prevention and treatment of hypertension and CCVDs and sheds light on the further studies in this regard. However, since most studies are *in vitro* and preclinical, there is a need for more in-depth researches and clinical trials to understand the potential of these substances.

## Introduction

It is universally acknowledged that the pathogenesis of cardiovascular and cerebrovascular diseases (CCVDs) is complex and long, which is an obstacle to the development of human health. In 2011, the United Nations recognised non-communicable diseases, including CCVDs, as the major health barriers for humans and developed plans to reduce the impact of these diseases (Mensah and Mayosi, 2013). According to WHO, 31% of the world's deaths in 2016 were from CCVDs. These causes included ischaemic heart disease (IHD), ischaemic stroke, haemorrhagic stroke, atrial fibrillation, peripheral arterial disease, aortic aneurysm, atherosclerotic, cardiomyopathy, and myocarditis, hypertensive heart disease, endocarditis, rheumatic heart disease, arrhythmias, etc (Kratz et al., 2017; Roth et al., 2017). Importantly, many common chronic CCVDs, such as heart failure, myocardial infarction, stroke, vascular dementia, and chronic kidney diseases, are closely related to hypertension (Sureda et al., 2017). However, hypertension is often caused by vascular lesions such as vessel wall thickening, vessel stenosis, vessel occlusion and endothelial cell injury (Mathieu et al., 2009; Farias, Pereira and Rosa 2010; Howlett 2014; Singh et al., 2017). For example, hypertension is more likely to cause IHD, stroke, cerebral haemorrhage and cerebral ischaemia in the elder population (Collaboration, 2002). Additionally, diabetic vasculopathy is concerned with vessel wall thickening and endothelial cell damage, which is the main cause of blindness, kidney failure, heart attacks, and stroke (Wimmer et al., 2019). Furthermore, hypertensive patients are also more likely to develop diabetes. (Sowers et al., 2001).

Remarkably, WHO encourages the use of traditional medicinal plants (TMPs) to improve various chronic diseases with increasing risk around the world. TMPs were shown to be beneficial for human health due to the richness of active compounds. Natural products, referring to small molecules derived from TMPs, also improve processes of biological metabolism through regulating the activity of enzymes in the body (Loai and Zohar, 2015; Briguglio et al., 2018). What’s more, TMPs have been used as food supplements to treat chronic diseases without prescription. Currently, TMPs and natural products are playing an indispensable part for improving human health as a complementary alternative therapy, although some side effects have been found. In the past two decades, scientists have explored the vasodilation of TMPs and natural products, and roughly explained their mechanism. These natural resources have also been proved to be potentially effective for the treatment of CCVDs in clinic and drawn greater attention, even in developed countries including the United States and Australia. (Delfan et al., 2014; Liu and Huang, 2016; Shaito et al., 2020). However, the vasodilatory activities, the underlying mechanism and the clinical efficacy of TMPs and natural products, had not been comprehensively over-reviewed.

This article reviews the underlying mechanism and the clinical efficacy of TMPs and natural products with vasodilation, and puts forward a view that TMPs and natural products can be used as adjuvants in the prevention and treatment of CCVDs. We hope this review will lay the foundation for an in-depth investigation on the development and utilization of nature resources in this filed.

## Search Strategy

This review aims to provide readers with a brief account of the main achievements in the field of vasodilation-based natural products and plant extracts from TMPs worldwide during the years of 1998–2020. To this end, the literatures from several databases including PubMed, Web of Science and Google Scholar were searched to obtain any studies evaluating TMPs and natural products with vasodilation. The data collected in this review were limited to English articles that primarily focused on the vasodilation and the underlying mechanisms of screening of TMPs and natural products.

## Vasodilation Mechanism of TMPs and Natural Products

**FIGURE 1:**
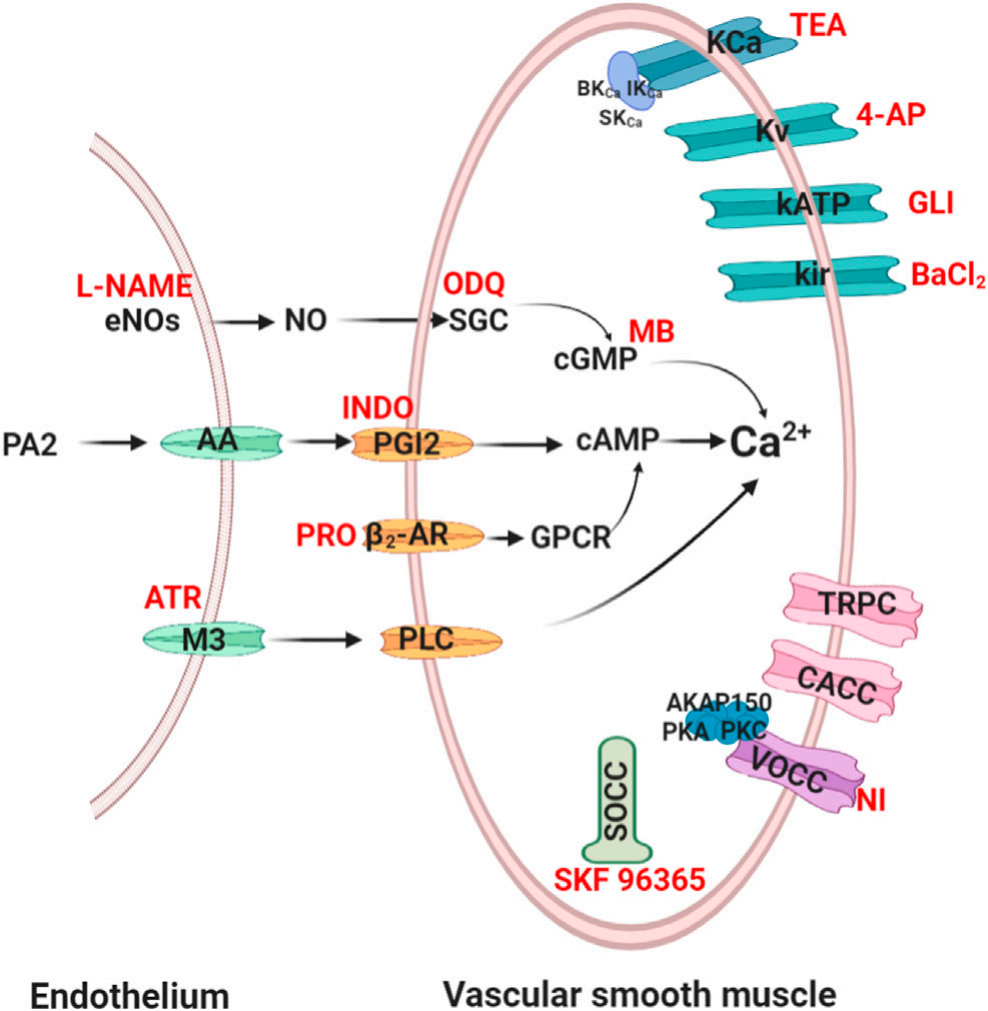
Routes of vasodilation mechanisms. Red words denote common blockers of the corresponding pathways. eNOs, endothelial nitric oxide synthase; SGC, soluble guanylyl cyclase; cGMP, cyclic 3′,5′-guanosine monophosphate; PA 2, phospholipase A 2; AA, arachidonic acid; PGI2, prostaglandin 2; cAMP, cyclic adenosine 3′, 5′-monophosphate; *β*2-AR, *β*2-adrenoreceptor; PLC, phospholipase C; L-NAME, nitro-L-arginine; ODQ, 1H- [1, 2, 4] oxadiazolo [4, 3-*α*] quinoxalin-1-one; MB, methylene blue; INDO, indomethacin; PRO, propranolol; ATR, atropine; NI, nifedipine; GLI, glibenclamide; 4-AP, 4-aminopyridine; TEA, tetraethylammonium.

The vascular tone changes of vascular smooth muscle cells (VSMCs) occurs through electromechanical and chemomechanical coupling and then acting on *α*-receptor, angiotensin-converting enzymes, angiotensin receptor or potassium/calcium channels. The detailed mechanism of action is as follows (Hill et al., 2001; Cole and Welsh, 2011). Figure 1 shows a brief description of the vascular tone changes of VSMs to support our understanding of vasodilatory mechanisms (Alexander et al., 2019a; Alexander et al., 2019b).

### Endothelium-Mediated Vasodilation Mechanism

Endothelial-derived relaxing factor (EDRF), including nitric oxide (NO) and prostacyclin from endothelial cells, plays an important role in vasodilation (Török, 2000; Tokoudagba et al., 2010).

#### NO Signalling Cascade-Mediated Vasodilation

NO is the main EDRF that induces relaxation of VSMCs. In addition, NO also plays a crucial role in CCVDs such as atherosclerotic disease (Malekmohammad et al., 2020). Endothelial nitric oxide synthase (eNOS) catalyses the production of NO, which diffuses into VSMCs and then enhances cGMP synthesis. cGMP activates dependent protein kinase to cause vasodilation by reducing the intracellular Ca^2+^ concentration (Bian et al., 2008). Therefore, to validate the eNOS/NO/sGC/cGMP/signalling pathway, L-NAME (non-selective NO inhibitor) is used to inhibit NO synthesis. ODQ (sGC pathways inhibitor) and MB (cGMP pathways inhibitor) are usually used to inhibit the sGC/cGMP pathway (Bian et al., 2008).

#### PGI2 Signalling Cascade-Mediated Vasodilation

Prostaglandin 2 (PGI2) is also a major EDRF, prostacyclin synthase-catalysed intermediate prostaglandin H_2_, and catalysed by cyclooxygenase (COX) for arachidonic acid synthesis. PGI2 activates adenylate cyclase (AC) to produce cAMP and then regulates dependent protein kinase to reduce the intracellular Ca^2+^ concentration (Ameer O. Z. et al., 2010). Indomethacin is commonly used to inhibit COX and PGI2 channels.

#### Muscarinic Receptor Signalling Cascade-Mediated Vasodilation

M3 receptor is a G*α*q-protein-coupled receptor (GPCR) that presents in endothelium. (Walch et al., 2000). Atropine is commonly used to block phospholipase-C signalling pathways by inhibiting M3.

### VSMCs Mediated Vasodilation Mechanism

The contraction of VSMCs mainly rely on Ca^2+^ influx via regulating ion channels (K^+^/Ca^2+^) or receptor channels. Ca^2+^ enter cells mainly through voltage dependent L-type Ca^2+^ channels (LTCC), store-operated calcium channels (SOCC) and transient receptor potential channels (TRPC). Of course, Ca^2+^ influx also relies on the regulation of Ca^2+^-gated Cl channels (CACC) (Brozovich et al., 2016). Moreover, K^+^ channels also changes of vascular tone by regulating extracellular Ca^2+^ influx.

#### *β*_2_-Adrenoreceptor-Mediated Vasodilation

*β*_2_-adrenoreceptor, a GPCR, only exists on the membrane of VSMCs. This receptor passes through the GPCR/AC pathway, which catalyses the breakdown of ATP into cAMP and subsequently causes vasodilation (Ch'ng et al., 2017). Propranolol, a nonselective *β*2-adrenergic receptor inhibitor, eventually causes vasoconstriction through inhibiting this channel.

#### Potassium Ion Channels Mediated Vasodilation

There are mainly four types of K^+^ channels in VSMCs: voltage-sensitive K^+^ channels (Kv), ATP-sensitive K^+^ channels (KATP), inward rectifier-type K^+^ channels (Kir) and Ca^2+^ activated K^+^ channels (KCa). KCa, widely appear in pulmonary artery smooth muscle cells, include large conductance Ca^2+^-dependent K channels (BK channels), intermediate-conductance Ca^2+^ activated K^+^ channel (IKCa) and small-conductance Ca^2+^ activated K^+^ channel (SKCa), and the BK channel was associated with the *β*-1 subunit (Brenner et al., 2000). The four channels were blocked by 4-aminopyridine (4-AP), glibenclamide, BaCl2, and tetraethylammonium (TEA), respectively (Su et al., 2014). Furthermore, other channels such as KCNQ subfamily, EAG (ether-à-go-go or KCNH) subfamily, Ca^2+^-activated Slo subfamily and Ca^2+^ and Na-activated SK subfamily were found in VSMCs (Brozovich et al., 2016; Barros et al., 2020).

#### Calcium Ion Channels Mediated Vasodilation

There are mainly three types of Ca^2+^ channels in VSMCs membranes, including voltage-operated calcium channels (VOCC) and receptor-operated calcium channels (ROCC). VOCC were regulated by membrane potential-dependent voltage, and ROCC bind to GPCR, SOCC and so on. LTCC, as a predominant VOCC, has been shown to be concerned with the A-kinase anchor protein 150 (AKAP150) and protein kinase C/A (Navedo et al., 2008). SOCC is mediated by the sarcoplasmic reticulum (SR) Ca^2+^ sensor stromal interaction molecule (STIM) and orai channels (Trebak, 2012). TRPC, including TRPC, TRPM, TRPV, TRPA, TRPP, and TRPML, are nonselective cation channels that carry receptor-operated Ca^2+^ currents (ROCs) triggered by phospholipase C (PLC)-catalyzed hydrolysis of phosphatidylinositol 4,5-bisphosphate [PI(4, 5)P2] (Mori et al., 2015). These channels may be also stimulated in store-operated manner, via tyrosine kinases, or by lysophospholipids, hypoosmotic solutions, and mechanical stimuli (Chen et al., 2020). Several TRPC, such as TRPC1, TRPC3, TRPC6, and TRP M4 were found to be involved in vasoconstriction and more sensitive than LTCC (Beech, 2005). Additionally, it was indicated that both SOCC and ROCC are concerned with TRPC family (McFadzean and Gibson, 2002). The blockers of VOCC and SOCC are nifedipine, SKF 96365 or Gd^3+^ respectively *in vitro* experiments (Xu et al., 2015; Alexander et al., 2019b).

## Traditional Medicinal Plants With Vasodilation *In Vitro* and Antihypertensive Activities *In Vivo*

**FIGURE 2:**
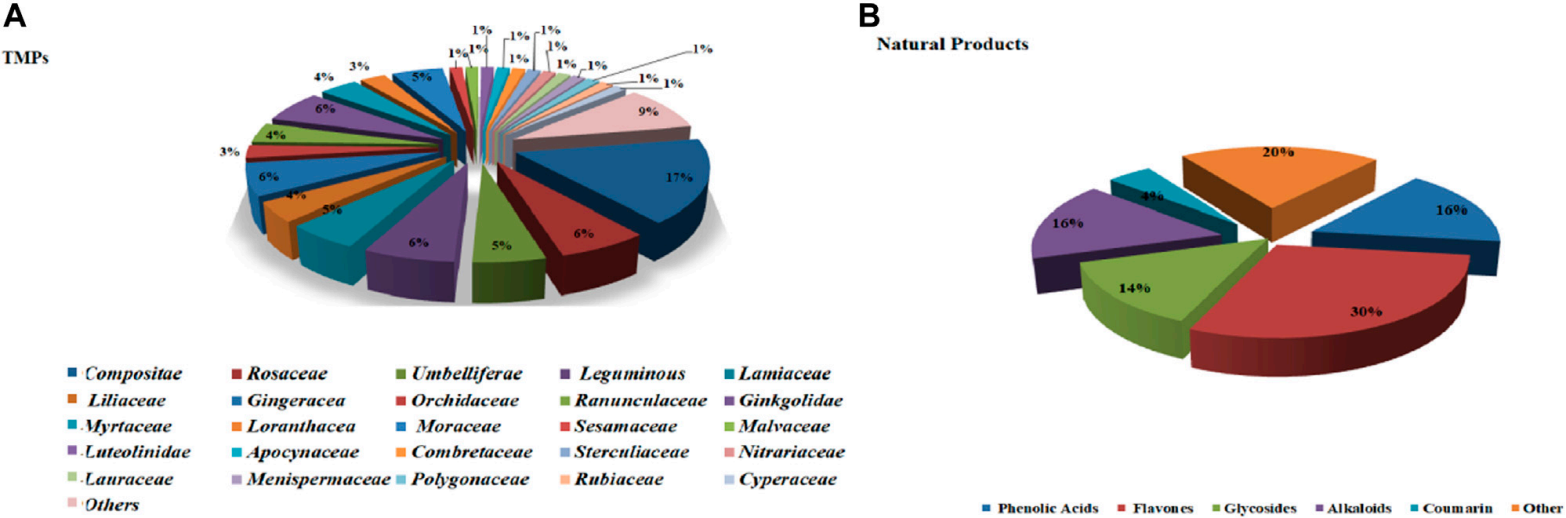
The ratio of TMPs and natural products. **(A)**: ratio of TMPs in this article (classified by family); **(B)**: ratio of natural products in this article (classified by compound type).

**FIGURE 3:**
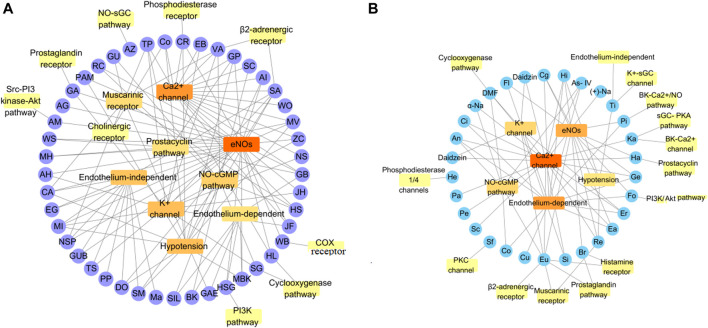
Some mechanisms of vasodilation caused by TMPs and natural products. **(A)** Vasodilation mechanism of TMPs; **(B)** Vasodilation mechanism of Natural Products. The vasodilatory function of TMPs was mainly concerned with the activation of eNOs, while the natural products were primarily associated with blockage of calcium channel. They also act on other pathways, including sGC-cGMP, potassium channels, muscarinic receptors, cyclooxygenase pathways, prostaglandin I2, etc.

This section describes the vasodilatory and antihypertensive effects of plant extracts. Most extracts act on complex pathways for vasodilation or hypotension in rats. Supplementary Table S1 shows the details of studies investigating vasodilation of TMPs. All the discussed TMPs, which were primarily from *Composite* (17%), were shown in Figure 2A. TMPs mainly displayed vasodilation by acting on eNOS and Ca^2+^ channels (Figure 3A). Most of TMPs, such as *V. amygdalina Del., T. procuinbens L., M. glomerata Spreng., K. galanga L.*, etc., have significant vasodilatory bioactivities *in vitro*, but the hypotensive efffect of only 40% of TMPs have been investigated *in vivo* at present, such as *G. procumbens, H. cere, Bidens pilosa Linn*, etc (Figure 3A). In addition, some TMPs have significant vasodilation but the mechanistic exploration was insufficient, such as *G. procumbens, M glomerata Spreng.*

### Compositae

#### *Erigeron breviscapus* (Vant.) Hand-Mazz.

*Erigeron breviscapus* (EB), as a traditional Chinese medicine, is commonly used for neuroprotection and vascular protection. EB induced relaxation on U44619 (Ca^2+^ agonists, EC_50_ 0.354 mg mL^−1^) pre-contracted aortic rings, which was abolished by glibenclamide (KATP inhibitor) or TEA (KCa channel inhibitor). However, this effect of EB was not affected by endothelial removal or ipiliocin (BKCa inhibitor), BaCl_2_ (Kir inhibitor) or 4-AP (Kv inhibitor). These results indicated that the vasodilatory activities of EB are mediated by Ca^2+^ and KATP channels, rather than endothelial cells or K^+^ channels (Pan et al., 2008).

#### *Vernonia amygdalina* Del.

*Vernonia amygdalina* Del. (VA) is used to treat hypertension in Malaysia. The ethanol extract of VA leaves (VAE) caused the relaxation of Phenylephrine (PHE, 1 μM)-pre-contracted aortic rings (EC_50_ 0.057 mg mL^−1^). However, its effects were significantly reduced by endothelial removal, TEA, 4-AP, BaCl_2_, glibenclamide, L-NAME (eNOS blocker), methylene blue (cGMP inhibitor), indomethacin (COX inhibitor), atropine (muscarinic receptor inhibitor) and propranolol (β_2_-adrenoreceptor inhibitor). Hence, the effect of VAE was related with the NO/cGMP/PGI2 pathways, Ca^2+^/K^+^ channels or β_2_-adrenergic receptor (Ch’ng et al., 2017). Additionally, VA (i.v. 10.0 mg kg^−1^) ultimately caused a reduction in blood pressure in SD rats (Taiwo et al., 2010).

#### *Tridax procuinbens* L.

The water extract of the leaf from *Tridax procuinbens* (TP) has been shown to reduce blood pressure, but its mechanism remains unclear. The data showed that TP could significantly relieve the contraction caused by PHE (0.1 μM) and K^+^ (60 mM). TP (10^−9^–10^−5^ M) also antagonised the Ca^2+^-induced vasoconstriction in a Ca^2+^-free context by high K^+^. The activity of TP was repressed by BaCl_2_ and apamin (K^+^ channels blockers), L-NAME, indomethacin, atropine, propranolol, and methylene blue; however, it was not affected by glibenclamide, TEA, 4-AP or ODQ (guanylyl cyclase inhibitor). Therefore, this action is intervened by endothelial cells and partial ion channels (Salahdeen et al., 2015).

#### *Artemisia herba* Alba Asso.

*Artemisia herba* Alba Asso (AH) is used to treat diabetes and hypertension in Morocco. AH relaxed the contraction elicited by noradrenalin (NE, 1 μM) in endothelium-containing aortas. However, its activity was significantly abolished by L-NAME (100 μM), endothelial removal, methylene blue (10 μM) and ODQ (50 μM) but not atropine (10 μM), TEA (5 mM), indomethacin (10 μM) or glibenclamide (10 μM). It was suggested that the vasodilation of AH occurs mainly through the activation of eNOS/SGC/cGMP pathways rather than K^+^ channels (Skiker et al., 2010).

#### *Bidens pilosa* L.

Previous studies have shown that the antihypertensive effect of *Bidens pilosa* (BP) extract is closely related to its vasodilatory activity. The extract of BP was shown to induce a concentration-dependent vasorelaxation of rat aortas pre-contracted by K^+^ (60 mM, inhibition rate 90% at 1.5 mg mL^−1^) and NE (1 μM, inhibition rate 88% at 1.5 mg mL^−1^) which was significantly inhibited by indomethacin or pyrilamine maleate (histamine-1 inhibitor) (Nguelefack et al., 2005). Furthermore, the extract of BP leaves (10, 20, and 30 mg kg^−1^) decreased SBP by 18.26, 42.5 and 30% in normotensive rats and by 25.77, 38.96 and 28.64% in SHR, respectively. Moreover, it also induced hypotension by 27, 34.13 and 18.73% in salt-loaded hypertensive rats, respectively (Dimo et al., 2003). These results indicated that the effect is concerned with vasodilation.

#### *Gynura procumbens* L. Merr.

*Gynura procumbens* (GP) has been shown to decrease blood pressure by inhibiting the angiotensin-converting enzyme. The present experiments showed that the aqueous extract of GP significantly reduced the contraction induced by angiotensin I and II in rat aortic rings, which was blocked by indomethacin (10 μM) or L-NAME (0.1 μM) (Poh et al., 2013). Moreover, the butanoic fraction of GP (2.5–5 mg mL^−1^) also released the PHE (1 μM)-/K^+^ (80 mM)-induced contractions. GP (i.v., 10–20 mg kg^−1^) also reduced mean arterial pressure in anesthetized rats (Hoe et al., 2011). Therefore, it was suggested that GP causes vasodilation through upregulating eNOS levels.

#### *Helichrysum ceres* S. Moore.

Ethnomedical evidences suggested that extracts from *Helichrysum* have anti-inflammatory and anti-allergic activities and are commonly used to treat renal and cardiopulmonary diseases. The researchers found that the ethanolic extract of Helichrysum ceres leaf (HCE) could relax atropine (1 µM)/NE (1 µM)/K^+^ (20, 80 mM)-induced contractions which was weakened by L-NAME (100 µM) in rat aortic. Furthermore, HCE could cause hypotension in normotensive rats or Dahl salt sensitive hypertensive rats (Musabayane et al., 2008). It was shown that activity of HCE is related to the eNOS levels.

#### *Mikania glomerata* Spreng.

*Mikania globerata* (MG) is mainly used to treat respiratory diseases in Brazil. The aqueous extracts and hydroalcoholic extract (HAE) of MG leaves significantly inhibited the histamine contractions in isolated guinea pig tracheas. HAE also induced relaxation in guinea pig tracheas pre-contracted by histamine (IC_50_ 0.34 mg mL^−1^), acetylcholine (IC_50_ 0.72 mg mL^−1^) or K^+^ (IC_50_ 1.41 mg mL^−1^). Moreover, the dichloromethane fraction of MG could relax the isolated mesenteric vascular or aorta in rats (Soares de Moura et al., 2002). But the mechanism of MG needs more exploration in the future.

#### *Flos chrysanthemi* Indici

The ethyl acetate extract from *Flos chrysanthemi* Indici (FCE, 9.4–150 mg/L) antagonized vasoconstriction induced by PHE (1 μM)/K+ (60 mM), which was significantly inhibited by endothelium removal, L-NAME (10^−4^ M), glibenclamide and methylene blue (10^−5^ M). But this effect of FCE was not affected by TEA, BaCl_2_, 4-AP, 5-HD or propranolol. In addition, FCE attenuated PHE-induced contraction in calcium-free or potassium-free solutions by upregulated NO levels in rat aortic. Overall, it was showed that FCE is in association with regulation of the NO/cGMP pathway and inhibition of KATP (Jiang et al., 2005).

#### Others

The hydroalcoholic extract of *Senecio nutans* sch. Bip and the aqueous extract of *Tanacetum vulgare* L were shown to relax the PHE/K^+^/NE-dependent contractions of rat aortic rings in an endothelium-dependent manner (Paredes et al., 2016), (Lahlou et al., 2008). Additionally, the dichloromethane extract of *Kaempferia galanga* L and the methanol extract from Stevia rebaudiana had shown antihypertensive activity in anaesthetised rats or SHR (Othman et al., 2006; Melis 1995). But the mechanism of these extracts is not sufficiently studied at present.

### Rosaceae

#### *Crataegus gracilior* J. B. Phipps.

Hawthorn is used worldwide as traditional medicines to treat CCVDS, such as *Crataegus gracilior* J. B. Phipps (Mexican hawthorn, MH). The aqueous extracts of the leaves and fruits of MH elicited relaxation of aortic rings treated by PHE (1 µM), and its methanol extract (EC_50_ 4.34 mg mL^−1^) had significant *vasodilator* effects (Hernández-Pérez et al., 2014). Additionally, the extract of Crataegus leaves and flowers caused relaxation in PHE (10 mM)-mediated rat aorta (IC_50_ 15.1 μg mL^−1^) or human papillary artery (IC_50_ 19.3 μg mL^−1^) from patients undergoing coronary artery bypass surgery. In short, the vasodilation of the extract is concerned with the eNOS pathway, but other pathways need to be further explored (Brixius et al., 2006).

#### *Fragaria x Ananassa* Duch.

The vasodilation activity of the aqueous extract of *Fragaria x ananassa* Duch (wild strawberry, WS) leaves attenuated NE-induced (0.1 µM) vasoconstriction in rat aortas which was suppressed by L-NAME or indomethacin. Therefore, similar to the hawthorn aqueous extract, the aqueous extracts could cause endothelium-dependent vasodilation which were correlated with the NO/COX pathways (Hernández-Pérez et al., 2014).

#### *Rubus chingii* Hu.

*Rubus chingii* (RC) is a commonly used traditional Chinese medicine that can improve renal function and treat polyuria. The ethanol extract of RC caused PHE (1 μM)-induced relaxation in rat aortas. However, this effect of RC was significantly attenuated by L-NAME (10 µM), ODQ (10 µM), diltiazem (10 μM, Ca^2+^ antagonist), wortmannin (0.1 µM, PI3-kinase inhibitor), thapsigargin (1 μM, Ca^2+^ modulator), Gd^3+^ (10 μM, Ca^2+^ modulator), 2-aminoethyl diphenylborinate (75 μM, Ca^2+^ modulator) and 4-AP (1 mM), rather than TEA (1 mM), glibenclamide (10 µM), indomethacin, atropine, and propranolol. This study indicated that RC induces vasodilation in an endothelium-dependent manner, mainly by activating the Ca^2+^/eNOS and the NO/sGC/cGMP/KV channels (Su et al., 2014).

#### *Aronia melanocarpa*

The high content of phenolic constituents of *Aronia melanocarpa* (AM) is characterized by a variety of biological activities. Researchers have found that AM juice caused potent endothelium-dependent relaxations in porcine coronary artery rings, which was markedly abolished by L-NAME, PP2 (Src kinase inhibitor) and wortmannin. This result showed that AM juice promotes NO levels in the coronary artery endothelium by activating the Src/PI3 kinase/Akt pathway. Its main active components may be conjugated anthocyanins and chlorogenic acid (Kim et al., 2013).

#### *Sorbus commixta* Hedl.

The cortex of this species has been used for antitussive purposes in oriental medicine. The methanol extract of *Sorbus commixta* cortex (SC) produced relaxation of the PHE-induced aorta which was attenuated by L-NAME, methylene blue, ODQ and endothelial removal except for indomethacin, glibenclamide, TEA, atropine or propranolol. Thus, its vasodilatory activity occurs through activation of the NO/cGMP pathway rather than blockage of KCa or KATP (Kang et al., 2005).

#### *Carum roxburghianum* (DC.) Kurz.

*Carum roxburghianum* (CR) exhibit vasodilatory and cardiac modulatory actions. The crude extract of CR (10–100 mg kg^−1^) induced decrease arterial blood pressure of rats. Meanwhile, CR also inhibited high K^+^ (80 mM)-/PHE (1 μM)-induced contractions in isolated rabbit aortas, which was not impaired by L-NAME. Therefore, CR extracts caused vasodilation might through antagonising Ca^2+^ channels, regulating NO levels and inhibiting phosphodiesterase (Khan et al., 2015).

### Umbelliferae

#### *Angelica dahurica* Bentham

*Angelica dahurica Bentham* (ADB) has been used for the treatment of CCVDs in Asia. The methanol extract of ADB (1 mg mL^−1^) resisted PHE (1 μM)-/K^+^ (60 mM)-induced contractions in aortic rings but not caffeine (opener of ryanodine-sensitive receptors) in a Ca^2+^-free context (Lee et al., 2015). But the vasodilatory activity of ADB needs further investigation.

#### *Ligusticum chuanxiong* Hort.

*Ligusticum chuanxiong* (Lc) have been widely used in the treatment of CCVDs in Asian countries. The CHCl_3_ extract of Lc could inhibit contraction induced by NE in aortic strips and abolished Ca^2+^-independent contractions evoked by 12-deoxyphorbol 13-isobutyrate in Ca^2+^-free medium containing EGTA (1 mM). Furthermore, Lc significantly inhibited NE-mediated extracellular regulated protein kinases 1/2 (ERK1/2) activation but not P38 mitogen activated protein kinases (MAPK) (Kim B. et al., 2004).

#### *Bupleurum fruticosum* L.

The studies have shown that *Bupleurum fruticosum* (BF) is beneficial to the heart and circulatory system. The chloroformic extract of the BF roots (0.1 mg mL^−1^) induced relaxation of rat thoracic aorta induced by NE or caffeine. This activity was blocked by cyclopiazonic acid (Ca^2+^- ATP blocker) (Testai et al., 2005).

#### *Coriandrum sativum* L.

Coriander (Co) is traditionally used for gastrointestinal diseases and CCVDs. Researchers have found that Co could resist K^+^ (80 mM)-induced contractions in the rabbit jejunum and PHE (1 μM)-/K^+^ (80 mM)-induced contractions in rabbit aortas. Meanwhile, Co (1–30 mg mL^−1^) caused hypotension in normal rats (Jabeen et al., 2009).

### Leguminous

#### *Glycyrrhiza uralensis* Fisch.

*Glycyrrhiza uralensis* (GU) is used in traditional Chinese medicine that displays a variety of bioactivities. The GU caused relaxation of rat aortic rings treated by PHE (1 µM)/K^+^ (80 mM) in endothelium-intact aortic rings. The vasodilatory activity of GU was weakened by L-NAME (10 µM), methylene blue (10 μM), indomethacin (10 μM), atropinee (1 µM), propranolol (1 µM), glibenclamide (10 μM) and 4-AP (1 µM) except for TEA (1 mM) or BaCl_2_ (10 μM) (Tan et al., 2017). Thus, GU may relax blood vessels through activating the NO/sGC/cGMP pathway rather than KCa or Kir.

#### *Albizia inopinata* G. P. Lewis.

The ethanol extract of *Albizia inopinata* G. P. Lewis (AI) could inhibit vasoconstriction induced by K^+^ (80 and 30 mM) or PHE (1 μM) (IC_50_ 54, 52, and 65 μg mL^−1^, respectively). Additionally, AI antagonised contractions caused in Ca^2+^-free medium induced by NE (1 μM) rather than caffeine (20 mM) in rat aortas (Pires et al., 2000). Furthermore, AI leaves abolished PHE (1 μM)/K^+^(80 mM)-induced vasoconstriction (IC_50_ 65 and 54 μg mL^−1^, respectively) which was repressed by endothelial removal or L-NAME (10 and 100 μM) but not atropine (1 μM) or indomethacin (10 μM). Moreover, AI leaves (20 mg kg^−1^) produced a significantly hypotensive effect and reduction in heart rate and cardiac output and total peripheral resistance in SHR (Maciel et al., 2004).

#### Others

The total alkaloids of *Sophora alopecuroids* L. (SA, 40 mg L^−1^) resisted concentrate induced by K^+^-/Ca^2+^ in rabbit aortas, while was not significantly affected by removal of endothelium, L-NAME, indomethacin or propranolol (Zhang et al., 2009). Seeds of *Securigera securidaca* (SS) were used for the improvement of hyperlipidaemia in Iranian medicine. The hydroalcoholic extract of SS could improve vascular endothelium-dependent relaxation and decrease lipid levels and peroxidation in a rat model fed a high-fat diet (Garjani et al., 2009).

### Lamiaceae

#### *Mentha x villosa* Hubs.

*Mentha x villosa* (MV) has anti-parasitic and tranquillising action and treatment of gastrointestinal diseases. Essential oil from MV induced significant and dose-dependent hypotensive and bradycardic responses, which were weakened by atropine (2 mg kg^−1^). Moreover, MV could attenuate PHE (1 μM) (IC_50_ 255 μg mL^−1^)-, prostaglandin F2-α (10 μM) (IC_50_ 174 μg mL^−1^)-/K^+^ (80 mM) (IC_50_ 165 μg mL^−1^)-induced contractions in rat aortic rings. This effect was abolished by endothelial removal, L-NAME (100 μM) or indomethacin (10 μM) but not atropine (1 μM) (Guedes et al., 2004). The results suggested that activity of MV is primarily associated with endothelial cells, but the role of ion channels is no clear at present.

#### *Ziziphora clinopodioides* L.

The CHCl_3_ extracts of *Ziziphora clinopodioides* L (ZC) inhibited PHE (1 μM, EC_50_ 0.27 g L^−1^)-/K^+^ (60 mM, EC_50_ 0.34 g L^−1^)-induced contractions in rat aortic rings, which was significantly decreased by 4-AP (1 mM) or endothelial removal, rather than glibenclamide (100 μm), iberiotoxin (10 nm) or thapsigargin (100 nm). In Ca^2+^-free solution, ZC also significantly inhibited vasoconstriction in K^+^/PHE pre-contracted rings (Senejoux et al., 2010). It was shown that activity of ZC is mainly associated with endothelial cells and Kv.

#### *Salviae miltiorrhizae* Bqe.

The roots of *Salvia miltiorrhiza* (SM) is used to treat CCVDS, such as angina pectoris and myocardial infarction in TCM. The aqueous extract of SM relaxed the NA-induced aorta which was abolished by L-NAME (100 μM), methylene blue (10 μM) and endothelium removal. Additionally, SM produced hypotensive response in normal rats through regulating release of angiotensin and bradykinin (Kamata et al., 1993). Moreover, SM caused hypotension in albino rats and rabbits, which was abolished by atropine and propranolol. Interestingly, low concentration of this extract, not higher concentration, induced vasodilation of the renal, mesenteric and femoral arteries (Lei and Chiou, 1986).

#### *Orthosiphon stamineus* Benth.

*Orthosiphon stamineus* in folk medicine is used in the treatment of hypertension and kidney stones. The methanolic extract of *Orthosiphon stamineus* (CF) relieved PHE (1 μM) (EC_50_ 2.21 μg mL^−1^)-/K^+^(60 mM) (EC_50_ 3.32 μg mL^−1^)-induced constriction of rat thoracic aorta which was resisted by L-NAME (10 μM, EC_50_ 17.8 μg mL^−1^), methylene blue (10 μM, EC_50_ 12.09 μg mL^−1^), TEA (1 mM, EC_50_ 24.35 μg mL^−1^), 4-AP (1 mM, EC_50_ 27.09 μg mL^−1^), BaCl_2_ (10 μM, EC_50_ 22.41 μg mL^−1^), glibenclamide (10 μM, EC_50_ 10.24 μg/mL^−1^) and propranolol (1 μM, EC_50_ 7.76 μg mL^−1^), rather than indomethacin (10 μM) (EC_50_ 4.62 μg mL^−1^) or ODQ (10 μM) (EC_50_ 3.87 μg mL^−1^). Thus, the vasodilatory activity of CF was in relationship with regulation of NO/cGMP pathway, muscarinic/*β*-adrenergic receptors and blockage of Ca^2+^ and K^+^ channels (Yam M. F. et al., 2016).

#### *Agastache mexicana* Ssp.

The dichloromethane extract of *Agastache mexicana* (AM) significantly relaxed NA (0.1 μM)-induced aortic contraction with endothelium (EC_50_ 174 μg mL^−1^) or without endothelium (EC_50_ 293 μg mL^−1^) which was significantly inhibited by TEA. In conclusion, this activity is correlated with endothelial cells or KCa (Flores-Flores et al., 2016).

#### *Marrubium vulgare* L.

The water extract of *Marrubium* (Ma) showed a potent inhibition on K^+^-induced rat aorta contractions and decreased SBP by improving endothelial function in SHR (El Bardai et al., 2003). But mechanisms of Ma needs more exploration *in vivo* or *vitro* in the future.

### Liliaceae

#### *Allium fistulosum* L.

Welsh onion (WO, *Allium fistulosum* L.) has been consumed to prevent CCVDs. The WO extracts, particularly green-leaf extracts (RG, 30–40 mg mL^−1^), could resist NE (30 nM)-induced contractions in aortic ring. The activity was abolished by L-NAME (100 μM), TEA (1 mM) and SQ29548 (TXA2-receptor antagonist, 10 μM) (Chen et al., 1999). The hydroalcoholic extract of onion peel relaxed vasoconstriction induced by K^+^/PHE which was not attenuated by endothelial removal, L-NAME (100 μM), methylene blue (10 μM) and indomethacin (10 μM) in rat thoracic aortas (Naseri et al., 2008). Thus, the activity of WO is independent of the eNOS/cGMP pathway and whether it acts on ion channels needs further investigation.

#### *Allium sativum* L.

The extracts of *Allium sativum* (AS) induce hypotension in hypertensive patients. AS could relieve NE (3 μM)-induced contractions in the aortic ring which was resisted by L-NAME (100 μM) and indomethacin (5 μM) (Takashima et al., 2017). Thus, the application about AS also needs to be supported by more research in CCVDs.

### Gingeracea

#### *Alpinia zerumbet* (Pers.) Burtt. et Smith.

The hydroalcoholic extract of Alpinia zerumbet leaves (AZ) could resist NE-induced contractions which was not suppressed by indomethacin, 4-AP or glibenclamide except for L-NAME and ODQ (de Moura et al., 2005). The essential oil of AZ (0.01–3,000 mg mL^−1^) also relieved PHE-induced contractions which was inhibited by L-NAME and endothelial removal but not TEA (500 mM) or indomethacin (10 mM) (Pinto et al., 2009). Thus, the activity of AZ is mainly concerned with endothelial cells.

#### *Curcuma comosa* Roxb.

Researchers have found that *Curcuma comosa Roxb* (CC) prevented the impairment of vascular relaxation by regulating the eNOS and ER-*α* protein levels in aorta of ovariectomised rats (Intapad et al., 2012). Additionally, CC promoted the phosphorylation of serine 1,177 in eNOS and serine 473 in Akt protein (Intapad et al., 2009). CC also improved the diastolic function of the aorta in hypercholesterolemic rats by activation of HSP70 and BCl-2 levels, improving activity of antioxidant enzymes (Kam et al., 2012).

#### *Curcuma longa* L.

The methanolic extract of *Curcuma longa* (i. v, CL, 10, 20 and 30 mg kg^−1^) could reduce blood pressure (2.0, 27.1, and 26.7%) and heart rate (5.8, 19.3, and 22.9%) in normal rats. CL (1–1,000 μg ml^−1^) also relieved PHE (10 μM)/K (80 mM) -induced aortic contraction, which was attenuated by glibenclamide, BaCl_2_, TEA or 4-AP. In addition, CL inhibited CaCl_2_ (1–30 mm) induced contraction in Ca^2+^ free medium. This suggested that CL relaxes blood vessels by blockage of K^+^ channels (Adaramoye et al., 2009).

#### Others

The methanol and water extracts of curcuma herbs such as *C. kwangsiensis* (1 mg mL^−1^), *C. phaeocaulis* (1 mg mL^−1^), *C. wenyujin* (1 mg mL^−1^), and *C. zedoaria* (1 mg mL^−1^) could relieve prostaglandin F-2α (6 μM)-induced contractions in aortic ring. Additionally, curcuma herbs such as *C. zedoaria* (3% wt/wt) could lower blood pressure and protect endothelial cells in SHR (Goto et al., 2005). Therefore, curcuma herbs, with acivities of invigorating blood circulation and eliminating stasis according to Chinese Medicine, may have significant potential for the prevention and treatment of CCVDs.

### Orchidaceae

#### *Orchis mascula.* ex L.

*Orchis mascula* (OM) is used to treat CCVDS in Pakistan and India. The OM extract inhibited PHE (1 μM)-/K^+^ (80 mM)-induced contractions in isolated rabbit aortas. OM (10 and 30 mg kg^−1^) also reduced SBP and improved endothelial function in SHR. Moreover, OM decreased TG, LDL-C levels in tyloxapol and high-fat diet-induced hyperlipidemia (Aziz et al., 2009).

#### *Dendrobium officinale Kimura.* et Migo.

*Dendrobium officinale* (DO) has been found to improve metabolic diseases including hypertension and diabetes mellitus. In addition, the extract of DO (3.1 g kg^−1^) significantly reduced SBP and mean arterial pressure in the hypertensive rats. Moreover, it reversed thoracic aortic thickening and endothelial cell apoptosis, decreased plasma ET-1/TXB2 levels and upregulated PGI2/NO levels (Liang et al., 2018).

### Ranunculaceae

#### *Paeonia suffruticosa* Andrew.

The methanolic extract of the root bark of *Paeonia suffruticosa* (PS) showed a vasodilation in rat aortas pre-contracted by PHE (0.3 μM, IC_50_ 16.8 μg mL^−1^). PS increased the endothelium and SOD function in rats fed a high-fat diet. Therefore, PM elicited vasorelaxant activity by protecting endothelial cells (Yoo et al., 2006).

#### *Nigella sativa* L.

*Nigella sativa* (NS, 2–14 mg mL^−1^) extract induced a dose-dependent relaxation in aortic rings treated by PHE (1 μM)/K^+^ (60 mM) which was abolished by diltiazem, TEA and glibenclamide except for L-NAME, indomethacin or ruthenium red (LTCC inhibitor). This finding was suggested that effect of NS is concerned with activating on K^+^ channels (Niazmand et al., 2014).

### *Ginkgo biloba* L.

*Ginkgo biloba* leaf extract (GB) has been clinically used to improve peripheral vascular disease in France and Germany. Researchers have found that GB produced a dose-dependent relaxation in aortic rings treated by NE, which was alleviated by L-NAME (100 μM), TEA (100 μM) and indomethacin (100 μM). In contrast, the effects of GB (3 mg mL^−1^) was strongly attenuated to 53% in Ca^2+^-free medium (Kubota et al., 2001), (Nishida and Satoh, 2003). Additionally, GB significantly reduced SBP in rats fed with 8.0% NaCl or SHR and potentiated the relaxation in response to acetylcholine in aortic (Kubota et al., 2006). However, GB was significantly increased serum alanine aminotransferase and hepatic CYP2B protein levels in aged SHR (Tada et al., 2008). It was suggested that the effect of GB was caused by Ca^2+^ channels inhibition and NO levels promotion, endothelial cells protection in SHR. Notably, terpenoids and flavonoids may be the main active components of GB (Nishida and Satoh, 2003; Seiichiro and Hiroyasu, 2004).

### Myrtaceae

#### *Syzygium guineense*

The hydroalcohol extract of the leaves of *Syzygium guineense* (SG) (50, 100, and 150 mg kg^−1^, orally) reduced systolic/diastolic blood pressure (6.9, 34.0, and 40.8 mmHg)/(5.0, 18.3, and 25.9 mmHg) in 1-kidney-1-clip hypertensive rat model. Moreover, the extract (70 mg kg^−1^) caused relaxation in aortas pre-contracted by K^+^ (80 mM), with a maximum relaxation of 56.22%. The relaxation mechanism was concerned with muscarinic or histamine receptors, KATP and COX/NO/cGMP pathway, independent of the endothelium system (Ayele et al., 2010).

#### *Myrciaria cauliflora*

Jabuticaba (*Myrciaria cauliflora*) hydroalcoholic extract (JH, 0.38–1.92 mg mL^−1^) resisted K^+^-/PHE-induced aortic ring contractions, which was weakened by TEA, glibenclamide and 4-AP. Therefore, JH might activates the K^+^/Ca^2+^ channels to cause vasodilation rather than SR Ca^2+^-ATPase or endothelium (Andrade et al., 2016).

#### *Pimenta dioica* L.

The aqueous extract of *Pimenta dioica* (EC_50_ 45 mg kg^−1^) decreased the blood pressure in SHR which was not related to ion concentration (K^+^,Na^+^,Ca^2+^, and Mg^2+^), *α*/*β*-adrenoceptor and cholinergic receptor (Suárez et al., 2000).

### Loranthaceae

#### *Loranthus ferrugineus* Roxb.

*Loranthus ferrugineus* Roxb could be successively fractionated by chloroform, ethyl acetate and n-butanol. n-butanol fraction of LFME (NBF-LFME) produced a significant inhibition of PHE-/K+-induced aortic ring contractions. Moreover, NBF-LFME lowered blood pressure more compared with the other fractions in normal rats. Thus, the effects of LFME were attributed to content of terpenoids in the n-butanol fraction (Ameer O. et al., 2010).

#### *Agelanthus dodoneifolius*

The ethanolic extract of *Agelanthus dodoneifolius* (AD, 0.01–10 mg kg^−1^, i,v.) could decrease the systolic and diastolic blood pressure in normotensive rats but not heart rate. Fruther, the extract was divided into 14 fractions (F1-F14). F4, contained most of the dihydropyranone dodoneine, produced the most effective activation (ED_50_ 160 μg mL^−1^) in NE-induced aortic rings contractions and reduced systolic and diastolic blood pressure by 56.9 and 81.6%, respectively in SHR (Ouedraogo et al., 2011). But the relationship between hypotensive activity and vasodilation of AD needs further investigation in the future.

### Moraceae

#### *Morus bombycis* Koidzumi

The ethanol extract of *Morus bombycis* koidzumi (MBK) exhibited vasodilatory effect (IC_50_ 3.9 μg mL^−1^) which was abolished by L-NAME or endothelial removal in rat aortas. Moreover, MBK extract (10, 30 and 100 mg kg^−1^) dose-dependently reduced SBP and attenuated liver lipid peroxidation and DNA-damage in SHR (Oh et al., 2007a). Therefore, the hypotensive activity of MBK is closely related to vasodilation, but the mechanism of vasodilation needs more exploration.

#### *Morus alba* L.

The major components of Mulberry leaves (ML) are polyphenols, flavonoids, carbohydrates, proteins and lipids. Researchers have found that ML could produce vasorelaxation of aortas treated by high K^+^ (60 mM) or PHE (1 μM) in arteries, which was abolished by ruthenium red (Xia et al., 2007). But mechanistic studies are notably imperfect such as endothelial cells and ion channels.

#### *Ficus sycomorus* L.

The extracts of *Ficus sycomorus* leaves (decoction, macerated and ethanol extract) exhibited a significant vasodilation in rat aortas (IC_50_ 1.27, 0.38, and 0.13 mg mL^−1^, respectively). But the effect of extract was inhibited by L-NAME except for ethanol extract (Ramdé-Tiendrébéogo et al., 2014). Thus, the vasodilatory potential of the extracts needs further exploration in the future.

#### *Humulus lupulus* L.

The extracts of *Humulus lupulus* (HL, 10^−9^–10^−2^ g L^−1^) inhibited NE (0.1 μM)-induced arterial ring contractions in sham-ovariectomised female rats. The vasorelaxation was strongly abolished by L-NAME (100 μM), indomethacin (10 μM) and thapsigargin (100 μM). The data suggested that vasodilation of HL is concerned with regulation of eNOS/COX levels and inhibition of Ca^2+^ channel (Figard et al., 2008).

### Other Sources

#### *Sesamum indicum* L.

The petroleum ether soluble fraction of the root extract of *Sesamum indicum* L (SIL, 180 μg mL^−1^) significantly inhibited PHE (2 μM)-/K^+^ (80 mM)-induced contractions (98.13 and 70.19%, respectively) in rat aortas. Additionally, which was abolished by L-NAME (300 μM) or methylene blue (10 μM) rather than propranolol (10 μM), atropine (1 μM), indomethacin (10 μM) or glibenclamide (10 μM). These results revealed that the vasodilation of SIL is chiefly mediated by endothelium-dependent pathway (Suresh Kumar et al., 2008).

#### *Hibiscus sabdariffa* L.

The methanolic extract of the calyces of *Hibiscus sabdariffa* L (HS) inhibited high K^+^ (80 mM)-/PHE (1 μM)-induced vasoconstriction in SHR. Meanwhile, the activity of HS was eliminated by atropine (1 μM), L-NAME (10 μM) or methylene blue (10 μM) except for indomethacin (10 μM). These results indicated that the effects of HS are likely mediated by the NO/cGMP pathway or Ca^2+^ channels (Ajay et al., 2007).

#### *Jasminum* Spp.

The ethanol extract of Jasmine flower (JF) caused a concentration-dependent relaxation in endothelium-intact rings treated by PHE (10 μM)/K^+^ (60 mM), which was decreased by L-NAME (3 mM), BaCl_2_ (1 mM), 4-AP (5 mM) and TEA (1 mM), expect for indomethacin (10 μM), and glibenclamide (10 μM). Additionally, JF inhibited the contraction of PHE after endothelium removal in Ca^2+^-free medium. The activity of JF was concerned with the NO levels or K^+^ channels (Yin et al., 2014).

#### *Hancornia speciosa* Gomes.

The extract of leaves from *Hancornia speciosa* Gomes (HSG) produced vasodilatation (pIC_50_ 5.6). This effect was suppressed by endothelial removal, L-NAME (100 μM), indomethacin (10 μM) or wortmannin (0.3 μM). In conclusion, HSG induced vasodilation in rat aortas possibly through PI3K activation (Ferreira et al., 2007).

#### *Pseuderanthemum palatiferum*

Water extracts from fresh leaves of *Pseuderanthemum palatiferum* (PP) caused the relaxation of NE-contracted endothelium-intact aorta rings (EC_50_ 81.0 μg mL^−1^), removal endothelium (EC_50_ 136.4 μg mL^−1^). Neither L-NAME nor atropine altered the vasodilation effect of the extract. The vasodilatory effect was partially dependent on endothelium rather than muscarinic receptor (Khonsung et al., 2011).

#### *Terminalia superba* Engl.et Diels

The aqueous, the methanolic and the methylene chloride extracts from the stem bark of *Terminalia superba* (TS) induced vasodilation on K^+^-/PHE-induced contractions in rat aortic rings. In contrast, the effect of TS was endothelium-dependent which was decreased by L-NAME (Tom et al., 2010).

#### *Guazuma ulmifolia* Lam.

The procyanidin fraction of *Guazuma ulmifolia* bark (GUB, 10 mg kg^−1^) decreased systolic arterial pressure and heart rate in hypertensive rats which was attenuated by L-NAME (31 mg kg^−1^). GUB reduced the contractions induced by NE (0.1 μM) in the aortic rings of normotensive (IC_50_ 35.3 ng mL^−1^) or hypertensive rats (IC_50_101 ng mL^−1^). This activity was attenuated by endothelial removal or L-NAME (30 μM) but not indomethacin (10 μM) or atropine (10 μM) (Magos et al., 2008). Thus, the potential of GUB is mainly associated with the endothelial rather than the PGI2 pathway.

#### *Nitraria sibirica* Pall.

The extract from the fruits of Nitraria sibirica Pall (NSP, 0.1–10 g L^−1^) produced vasodilation in PHE (1 μM)-/K^+^ (60 mM)-induced pre-contracted aortic rings which was significantly suppressed by endothelial removal, L-NAME (100 μM), atropine (1 μM) and charybdotoxin (30 nM, K^+^ channel blocker) plus apamin (30 nM). Furthermore, NSP (i.v. 1, 5, 10, and 20 mg kg^−1^) induced hypotensive effect in SHR and Wistar rats (Senejoux et al., 2012). But the relationship between the hypotensive activity and the vasodilation needs further exploration.

#### *Persea americana* Mill.

The aqueous extract of *Persea americana* Mill (PAM) abolished the positive inotropic and chronotropic responses induced by NE (10^−10^–10^−5^ M)/Ca^2+^ (5–40 mM) in guinea pig atrial muscle. However, this effect was markedly inhibited by L-NAME (10 μM). Furthermore, PAM (i.v. 25–400 mg kg^−1^) significant reduced blood pressure and heart rates in normotensive and hypertensive rats (Ojewole et al., 2007).

#### *Stephania abyssinica* Walp.

*Stephania abyssinica* Walp (SA) is used to treat arterial hypertension in west region of Cameroon. A previous study indicated that aqueous (ASAW) and methanol (MSAW) extracts from fresh leaves of S. abyssinica exhibited vasorelaxation by K^+^-induced contractions (EC_50_ 0.16, 0.35 mg kg^−1^) in aortic rings. ASAW (EC_50_, 0.18 mg kg^−1^) also resisted contracted by PHE in aortic rings which was affected by TEA, glibenclamide, and propranolol but not L-NAME. These results indicated that the vasodilation of ASAW is mediated by Ca^2+^/KATP channels (Nguelefack et al., 2015).

#### *Fagopyrum esculentum* Moench.

The hot-water extract of *Fagopyrum esculentum* (FE) evoked a significant vasodilation in rat aortic rings contracted by PHE (1 μM, EC_50_ 2.2 mg mL^−1^) or K^+^ (50 μM, EC_50_ 1.9 mg mL^−1^). Moreover, the acidic fraction of FE (EC_50_ 0.25 mg mL^−1^) had markedly stronger effect which was weakened by endothelial removal or L-NAME (100 μM). These results suggested that acidic partial vasodilation is mediated by the NO/cGMP pathway (Ushida et al., 2008).

#### *Uncariae ramulus* et Uncus.

The alcohol extract of *Uncariae ramulus* et Uncus displayed a vasodilatory effect. The underlying mechanism consists of Ca^2+^ channel blockage and endothelium-dependent. The alkaloids and tannins might be the main active components. Additionally, Uncaria sinensis hexane extracts (HEUS), ethyl acetate extracts (EAEUS) and methanol extracts (MEUS) had a protective effect against photothrombotic ischaemic injury in mice. HEUS (10, 50, and 100 mg kg^−1^) also reduced infarct volume and edema compared with EAEUS and MEUS. However, HEUS did not reduce eNOS levels of brain infarction in mice, suggesting that the protective effect of HEUS is primarily concerned with endothelium (Yuzurihara et al., 2002), (Goto et al., 2000).

#### Capparis aphylla

The extract of *Capparis aphylla* (CA) could inhibit PHE (1 μM) (EC_50_ 0.1 mg mL^−1^)/K+ (80 mM) (EC_50_ 1.22 mg mL^−1^)-induced contractions in rabbit aortic rings, which was abolished by endothelium removal, L-NAME and atropine. Moreover, CA (3–100 mg kg^−1^, i.v.) reduced mean arterial pressure in normotensive rats, which was partially blocked by atropine (2 mg kg^−1^) (Shah and Gilani, 2011). Thus, the activity of CA may be associated with activation of muscarinic receptors or eNOS.

#### Others

The aqueous extract of *Rheum undulatum* L. (Ru) could alleviate PHE-induced constriction in rat aortic, which was attenuated by endothelial removal, L-NAME, methylene blue and ODQ. These results suggested that activity is mediated by NO/cGMP pathways (Kim H. H. et al., 2004). The aqueous extract of Echinodorus grandiflorus (EG, 0.1–10 mg) significantly induced renal vasodilation in rabbits treated by NE-construction which was attenuated by L-NAME (100 μM) and methylene blue (20 μM). However, its activity was not affected by charydbotoxin (100 nM, Ca^2+^ blocker) or glibenclamide (3 μM) (Tibiriçá et al., 2007). The extracts of *Maytenus ilicifolia* (MI) leaves reduced the mean arterial pressure and heart rate in anaesthetised rats which was significantly reduced by L-NAME, methylene blue, ODQ, TEA, 4-AP and glibenclamide except for atropine, propranolol (Crestani et al., 2009). The extract of *Globularia alypum* (GA) also relieved diastolic PHE (2–4 ng mL^−1^)-induced mesenteric artery contractions in rats, which was inhibited by endothelial removal or atropine, but not indomethacin (10 μM) or L-NAME (100 μM) (Chokri et al., 2012). The extract of *Gmelina arborea* hexane leaves (GAE) produced vasodilatory effects in PHE (1 μM)-induced contractions which was reduced by L-NAME (2 μM), indomethacin (2 μM) and glibenclamide (2 μM) (Wansi et al., 2012).

## Natural Products With Vasodilation *In Vitro* and Antihypertensive Activities *In Vivo*

This section summarized the vasodilation of natural compounds. Some compounds have significant vasodilation (EC_50_/IC_50_ < 10 μM), whose mechanism are related to multi-pathways (Supplementary Table S2). The natural products, mainly flavones (30%), were shown in Figure 2B. Importantly, they showed vasodilation mainly by acting on Ca^2+^ channels and eNOS (Figure 3B).

**FIGURE 4:**
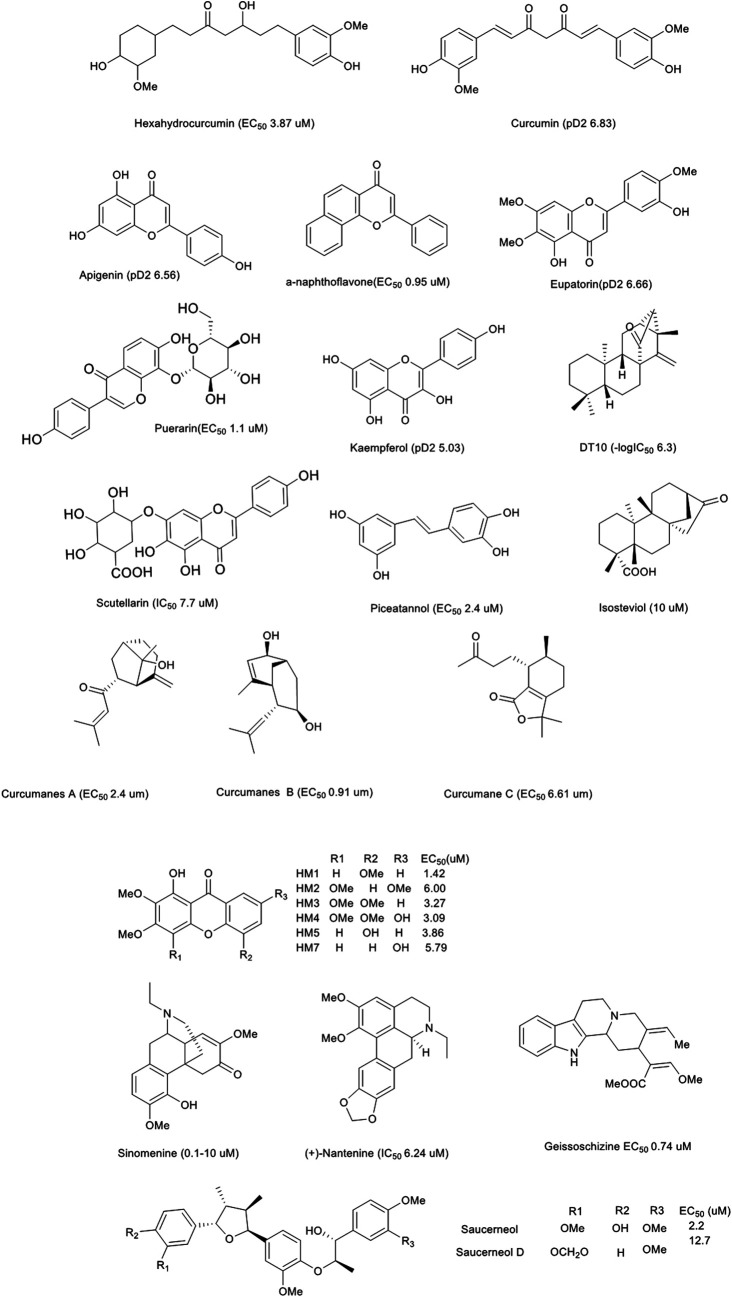
Natural products with significant vasodilation effect.

Additionally, we briefly analysed the structure-activity relationship of the compounds with remarkable functions, as shown in Figure 4. Among the phenolic acids, hexahydrocurcumin and curcumin had the prominent vasodilatory effects. The difference in bioactivity may be related to the number of double bonds. All compounds, specifically flavones, displayed the most excellent potential. To summarise, the vasodilation of these compounds was related to the number and position of double bonds, carbonyls, phenolic hydroxyl groups and methoxy groups. Furthermore, some compounds, such as chrysin glucoside, tilianin, reticuline and hirsutine also exhibited hypotension *in vivo* (Figure 3B).

### Flavones

#### Apigenin

Apigenin (Ap) was a flavonoid in the Chinese herbal medicine Flos Chrysanthemi that displays anti-hypertensive and anti-inflammatory activities. Ap markedly reduced rat thoracic aorta contractions induced by pyrogallol or acetylcholine (pD2 6.56, 5.31), which was weakened by L-NAME rather than aminoguanidine or indomethacin. Additionally, Ap significantly reduced blood pressure of in hypertension rat by ameliorating NO levels and nitrite urinary excretion (Jin et al., 2009).

#### ***α***-Naphthoflavone

*α*-Naphthoflavone (*α*-NF) induced relaxation in PHE-induced aortas (EC_50_ 0.95 μM), which was significantly inhibited by endothelial removal, L-NAME or methylene blue. In HUVECs, *α*-NF also activated NO channels which was blocked by SKF 96365 (Ca^2+^ channel blockers) and Ni^2+^ (Ca^2+^ channel blockers). Thus, the vasodilatory activities of *α*-NF might be mediated by the Ca^2+^ channels and the NO/cGMP pathway (Cheng et al., 2003).

#### Formononetin

Formononetin (Fo), as a methoxylated isoflavone, enhanced NO levels or endothelial cell function and also induced vasodilation by K^+^ pre-contractions in rat aortas (EC_50_ 107.2 μM). This action was reduced by endothelial removal and L-NAME. Moreover, the vasodilatory activity was concerned with the phosphatidylinositol 3-kinase/protein kinase B (PI3K/Akt) pathway (Li et al., 2018).

#### Puerarin

Puerarin (Pu) is the main isoflavone found in Pueraria lobata, which has been used for CCVDs. Pu (10^−10^−10^−8^ M) induced relaxation in PHE-induced aortas through promoting HO function in endothelial removal. Additionally, Pu caused relaxation of aortic rings contracted with NE (1 μM) (EC_50_ 1.1 μM), which was significantly repressed by iberiotoxin (50 nM, BKCa blocker). Pu also alleviated high glucose-induced endothelium-dependent vascular dysfunction in rat aortic rings (Meng et al., 2009), (Sun et al., 2007). Thus, the activities of Pu are concerned with endothelial cells or BKCa.

#### Hesperetin

Hesperetin (He) was shown to relieve NE (1 μM)-/K+ (60 mM)-induced contractions (IC_50_ 62.8, 62.2 μM). The effects were not inhibited by endothelial removal, glibenclamide (10 mM), TEA (2 mM) or nifedipine (0.1 μM). Moreover, He inhibited calmodulin-activated PDE1 and PDE4 isolated from bovine aortas (IC_50_ 74, 70 μM) (Orallo et al., 2004).

#### 5, 7-Dimethoxyflavone

In Thai traditional medicine, Kaempferia parviflora is used to treat CCVDs, including hypertension, asthma or diarrhoea. 5, 7-dimethoxyflavone (DMF, 1–100 μM), which was the primary component of Kaempferia parviflora, caused relaxations in aortic rings pre-contracted by methoxamine. This effect was abolished by endothelial removal, L-NAME (300 μM), indomethacin (10 μM), TEA (5 mM), glibenclamide (10 μM), 4-AP (1 mM), BaCl_2_ (10 μM) or ODQ (10 μM). Therefore, DMF-induced relaxation was mediated through the activation of the NO/cGMP/COX pathways and the inhibition of Ca^2+^ channels (Tep-Areenan et al., 2010).

#### Eupatorin

Eupatorin (Eu) is the primary component in Orthosiphon stamineus, used in Malaysia to treat hypertension. Eu produced relaxation of aortic rings pre-contracted by PHE in intact endothelium. This effect was reduced by L-NAME (pD2 4.60), methylene blue (pD2 6.05), ODQ (pD2 5.84), indomethacin (pD2 6.27), TEA (pD2 6.09), 4-AP (pD2 6.34), BaCl_2_ (pD2 6.47), atropine (pD2 6.36) and propranolol (pD2 6.49). Overall, the effect of Eu was related to activation of the NO/SGC/cGMP pathway, regulation of muscarinic/β-adrenergic receptor and inhibition of K^+^ channels (Yam M. et al., 2016).

#### Scutellarin

Scutellarin (Sc), obtained from Erigeron breviscapus Hand. Mazz, has been used to treat CCVDs such as hypertension. Sc aroused relaxation in rat aortic rings pre-contracted by NE (1 μM, IC_50_ 7.7 μM), which was not influenced by TEA (10 mM), glibenclamide (10 μM), atropine (100 nM), propranolol (1 μM), indomethacin (10 μM) or L-NAME (100 μM). Additionally, Sc inhibited the increase of intracellular Ca^2+^ induced by NE and had no effect on phorbol-12, 13-diacetate-induced contractions in Ca^2+^-free solution (Pan et al., 2008). It was shown that the activity of Sc may be through acting on ion channels rather than endothelial cells.

#### Daidzein/Daidzin

Daidzein and daidzin were shown to produce relaxation in aortas pre-contracted by U46619 (100 nM) (IC_50_ 20 and 140 μM, respectively), which was weakened by glibenclamide (1 μM), TEA (100 mM), iberiotoxin (100 nM), 4-AP (1 mM) or BaCl_2_ (100 μM). Daidzein-induced vasodilation of rat cerebral basilar arteries was partially dependent on BKCa channels in VSMCs (Zhang H.-T. et al., 2010). Therefore, the effects of daidzein and daidzein were concerned with inhibition of K^+^ channels (Sun et al., 2007).

#### Kaempferol

Kaempferol (Ka) produced relaxation in pulmonary arterial rings pre-contracted by PHE (1 µM) (pD2 5.03), which was not affected by glibenclamide, BaCl_2_, 4-AP (1 mM), L-NAME or indomethacin. However, this activity was repressed by TEA (10 mM) and ODQ. Therefore, Ka caused vasodilation through blocking BKCa/LTCC or regulating the sGC/PKA pathway (Mahobiya et al., 2018).

#### Baicalein/Luteolin

Baicalein and luteolin were shown to significantly alleviate insulin resistance (IR)-induced SBP elevation which were inhibited by bisphenol A diglycidyl ether in rats fed fructose for 12 weeks. Meanwhile, they also reduced excessive vasoconstriction of PHE/K^+^ in IR animals through elevating NO/ROS (reactive oxygen species) levels (Huang et al., 2004).

#### Genistein

Genistein (4′,5,7-trihydroxyisoflavone) is naturally occurring flavonoid found in the Leguminosae plant. Studies had shown that genistein elicited vasodilatory, anti-thrombotic and anti-atherosclerotic. This vasodilation was closely related to regulation of eNOS/angiotensin levels or inhibition of K^+^/Ca^2+^ channels (Sureda et al., 2017).

#### Others

Hydroxy-2,3,5-trimethoxy-xanthone (HM-1, EC_50_ 1.42 μM), 1-hydroxy-2,3,4,7-tetramethoxy-xanthone (HM-2, EC_50_ 6.00 μM), 1-hydroxy-2,3,4,5-tetramethoxy-xanthone (HM-3, EC_50_ 5.27 μM), 1,7-dihydroxy-2,3,4,5-tetramethoxy-xanthone (HM-4, EC_50_ 3.09 μM), 1,5-dihydroxy-2,3-dimethoxy-xanthone (HM-5, EC_50_ 3.86 μM) and 1,7-dihydroxy-2,3-dimethoxy-xanthone (HM-7, EC_50_ 5.76 μM) caused vasodilation in coronary arteries pre-contracted by 5-hydroxytryptamine (5-HT, 1 μM). Furthermore, removal of the endothelium decreased the vasodilation of HM-1 and HM-7 but did not affect HM-2, HM-3, HM-4 or HM-5 (Wang et al., 2009). The number of methoxy groups (HM 1, HM 2) and the substitution position of hydroxyl groups (HM 5, HM 7) had significant effects. In addition, Floranol (Fo) was shown to induce vasodilation pre-contracted by PHE (0.1 μM) (IC_50_ 19.9 μM), which was not affected by L-NAME or endothelium removal (Lemos et al., 2006).

### Alkaloids

#### Harmaline

Harmaline (Ha) has shown hypotensive activity *in vivo*, but the mechanism is no clear at present. Researchers had found that Ha (3, 10, and 30 μM) induce relaxation in aortas pre-contracted by NE/K^+^, which was significantly suppressed by L-NAME, indomethacin, prazosin (α-adrenoreceptors blocker) or diltiazem. Thus, the effect of Ha was related with activation of the prostacyclin or eNOS pathway (Berrougui et al., 2006).

#### Reticuline

Reticuline (Re) resisted contractions induced by PHE (1 μM) and K^+^ (80 mM) (IC_50_ 40 and 240 μM, respectively). Additionally, Re (i.v. 5, 10 and 20 mg kg^−1^) produced hypotensive effect in normotensive rats, which was attenuated by L-NAME (20 mg kg^−1^) or atropine (2 mg kg^−1^). These results suggested that Re reduced blood pressure by activating the muscarinic or eNOS receptors, and blocking Ca^2+^ channels (Dias et al., 2004).

#### Sinomenine

Sinomenine acutum Rehder has been used in the treatment of rheumatoid arthritis in China and its extract is also found to have vasodilator activity. Sinomenine (Si, 0.1–10 μM), as the mainly compound from Sinomenine acutum Rehder, produced relaxation in aortic rings pre-contracted by PHE (10 nM) or K^+^ (40 mM) which was attenuated by glibenclamide. Similarly, Si (1–100 μM) also reduced Ca^2+^ concentration in aortic smooth muscle (A7r5) cells induced by PHE (1 μM) or K^+^ (40 mM), which was eliminated by glibenclamide. Moreover, Si (i.v. 2.5–10 mg kg^−1^) decreased SBP in SHR (Lee et al., 2007). Thus, the activity of Si was concerned with activation of KATP.

#### (+)-Nantenine

(+)-Nantenine (Na), isolated from Nandina domestica, was widely used for the treatment of asthma, uterine bleeding and diabetes. Na could relieve contractions induced by NE (IC_50_ 6.24 μM) or high K^+^ (60 mM, IC_50_ 5.23 μM) in rat aortic rings, which was not modified by endothelial removal, glibenclamide (10 μM) or TEA (2 mM) (Orallo and Alzueta, 2001). The activity of Si was concerned with acting on KATP or endothelial cells.

#### Cassiarin A

The leaves of *Cassia siamea* were used for the treatment of hypertension in traditional folk medicine. Cassiarin A (Ca), isolated from Cassia *siamea*, was shown to induce relaxation in mesenteric arteries pre-contracted with PHE (1 μM, EC_50_ 6.4 μM), which was significantly reduced by endothelial removal, L-NAME (100 μM), ODQ (10 μM), TEA (1 mM) and iberiotoxin (100 nM) rather than indomethacin (10 mM), glibenclamide (10 mM) and 4-AP (1 mM). These results suggested that Ca activity is mediated by NO and BKCa channels (Matsumoto et al., 2010).

#### 2-Benzyl-5-Hydroxy-6-Methoxy-3,4-Dihydroisoquinolin-1-One

Isoquinolinone alkaloids have antihypertensive, antiarrhythmic and other activities. Researchers have found that 2-Benzyl-5-hydroxy-6-methoxy-3, 4-dihydroisoquinolin-1-one (ZC2) relax mesenteric artery segments pre-contracted by KCl (pEC_50_ 4.56), PHE (pEC_50_ 5.39) or U46619 (pEC_50_ 4.67), which was not modify by endothelial removal or glibenclamide (10 μM). Thus, ZC2 induced vasodilation through inhibiting VOCC and ROCC (Xu et al., 2012).

#### Geissoschizine Methyl Ether/Hirsutine

Geissoschizine methyl ether (Ge) and hirsutine resisted NE (10 nM)-induced contractions (EC_50_ 0.74, 10.6 μM) which were abolished by endothelial removal or L-NAME (100 μM). Furthermore, hirsutine reduced the SBP and heart rate of SHR (Yuzurihara et al., 2002). These results were suggested that the vasodilatory activity of NE is closely related to endothelial cells.

#### Piperine

Piperine (Pi), as main compound in Sahatsatara formula, had been demonstrated to have hypotensive effects in L-NAME-induced endothelial dysfunction rats. Moreover, it relaxed the thoracic aorta and has vascular protection effects against hypertension in rats (Booranasubkajorn et al., 2017).

#### Uncarialin A

*Uncaria rhynchophylla* Miq. ex Havil has been used to treat hypertension and epilepsy in clinics. Uncarialin A, separated from the hooks of *U. rhynchophylla*, resisted PHE-induced contractions (IC_50_ 0.18 M) independent of endothelial cells. Additionally, Uncarialin A reduced concentration of Ca^2+^ in VSMCs by inhibiting the LTCC subunit *α*-1c (Cav1.2) (Yun et al., 2020).

#### 8-Oxo-9-Dihydromakomakine

8-Oxo-9-dihydromakomakine (Di), separated from the leaves of Aristotelia chilensis, produced dose-dependent relaxation of aortic rings pre-contracted by PHE (1 μM)/KCl (60 mm), which was inhibited by TEA and BaCl_2_. It was suggested that the activity of Di is related to reduction intracellular Ca^2+^ concentration by inhibiting KCa or Kir (Cifuentes et al., 2018).

#### Curine

Curine could inhibit contractions induced by KCl and Bay K8644 in rat aortas and decreased intracellular Ca^2+^ concentration in VSMCs. The activity was not abolished by 3-isobutyl-1-methylxanthine (phosphodiesterase inhibitor), dibutyryl cyclic AMP (protein kinase A activator) or 8-br-cyclic GMP (protein activator kinase G) (Medeiros et al., 2011). It was indicated that potential of curine is associated with endothelial cells and ion channels.

### Phenolic Acids

#### Ethyl Rosmarinate

Ethyl rosmarinate (Er), as a major component of Rosmarinus officialis and Hyptis suaveolens, has cardioprotective activities. Er produced relaxation pre-contracted by PHE (10 μM) (pD2 4.42) or K^+^ (80 mM) (pD2 4.56) in endothelium-denuded rings. However, the effect was not abolished by TEA (5 mM), glibenclamide (10 μM), BaCl_2_ (1 mM) and ODQ (1 μM) except for 4-AP (1 mM). Er also reduced the contractions induced by deoxyepinephrine (10 µM) and caffeine (20 mM) in a Ca^2+^-free solution and inhibited extracellular Ca^2+^ influx. Therefore, the vasodilation of Er was mediated by endothelial cells or Kv (Wicha et al., 2015).

#### Paeonol

Paeonol (Pa) is the main component of the Chinese herbs *Paeonia suffruticosa* Andr. and *Cynanchum paniculatum* (Bunge) Kitagawa. Pa displayed anti-ischemia reperfusion injury, antihypertensive, anti-platelet aggregation, scavenges oxygen free radicals, anti-atherosclerosis and anti-VSMCs proliferation activities. Researchers have found that Pa relaxed PHE-induced isolated rat aortic rings (EC_50_ 290 μM). Additionally, Pa significantly inhibited vasoconstriction induced by angiotensin II, prostaglandin F-2α, 5-HT, dopamine, vasopressin and endothelin-1. Moreover, the activity of Pa was not affected by L-NAME, ODQ, propranolol, glibenclamide, TEA and BaCl_2_ in the rings. Therefore, the vasodilatory effect of Pa was in relationship with regulation of Ca^2+^ channels (Li et al., 2010).

#### Hexahydrocurcumin/Curcumin

Curcumin (Cu) was the main active component of the roots of *Curcuma longa* L. Hexahydrocurcumin (HCC) was a reduction product of Cu and metabolites in mice or humans. HCC has a wide variety of pharmacological effects, including anti-inflammatory, anti-viral, anti-fungal, anti-oxidant and anti-cancer effects. Moreover, HHC relaxed endothelium-intact aortic rings pre-contracted by PHE (10 μM, EC_50_ 3.87 μM) or K^+^ (80 mM, EC_50_ 95.12 μM). This agent relieved the CaCl_2_-induced contractions in K^+^ solutions and also suppressed the contractions induced by PHE/caffeine in Ca^2+^-free solutions. Additionally, HHC relieved phobal-12-myristate-13-acetate (PMA, activator of PKC) pre-contracted aortic rings (EC_50_ 93.36 μM). Collectively, it was suggested that the effect of HHC is related to endothelium cells, VOCC, ROCC and PKC-mediated Ca^2+^ channels (Moohammadaree et al., 2015). Cu (pD2 6.83) also relaxed the aortic rings pre-contracted by PHE (1 μM). Further, Cu reversed the vasodilatory dysfunction induced by high glucose (44 mmol/L) by improving HO-1 function in aortic rings. However, the activities were resisted by protoporphyrin IX zinc (1 μM, inhibitor of HO-1) or methylene blue (1 μM) (Sasaki et al., 2003).

#### Sodium Ferulate/Ferulic Acid

Ferulic acid (Fa) was the main component of Radix Angelicae Sinensis and Rhizoma Chuanxiong. Studies have shown that sodium ferulate (Sf) or Fa displayed anti-platelet aggregative, anti-inflammatory, antioxidative activities. Sf relaxed the aortic rings pre-contracted with PHE/K^+^ (pD2 2.7 and 2.6, respectively), which was no affected by TEA, glibenclamide, 4-AP and BaCl_2_. Sf also inhibited contraction in K^+^/PE/PMA pre-contracted rings in Ca^2+^-free solution (Chen G. et al., 2009). Moreover, Fa decreased superoxide anion levels in SHR aortas and improved acetylcholine-induced vasodilation in SHR but not in WKY rats (Suzuki et al., 2007). This activity was related to the methoxy modified 3-position in the benzene ring and 2-propylene (Fukuda et al., 2015).

#### Ellagic Acid

Ellagic acid (Ea), a polyphenolic compound, has anti-hypertensive, anti-diabetic, anti-oxidantive, anti-inflammatory and anti-hyperlipidaemia effects. Ea relaxed the aortic pre-contracted by PHE (pD2 5.60), which was partially abolished by endothelium removal and L-NAME rather than indomethacin. Therefore, the activity of Ea was related to endothelium cells and Ca^2+^ channels (Yılmaz and Usta, 2013).

### Glycosides

#### Astragaloside IV

*Astragalus* membranaceus has been used to treat and prevent CCVDs such as haemorrhagic stroke and viral myocarditis. Astragaloside IV (As-IV), the main component of A. membranaceus, has anti-cardiac hypertrophy, anti-inflammatory and anti-oxidant activities. As-IV could antagonise contractions induced by PHE/K^+^ in aortic rings from normal rats and SHR. Similarly, As-IV attenuated the vasoconstriction induced by angiotensin II or PHE in presence of perivascular adipose tissue (Zhang et al., 2006). This activity of As-IV was abolished by L-NAME or ODQ rather than 7-nitroindazole (neuronal eNOS inhibitor). Therefore, this vasodilatory was associated with blockage of calcium channel, and activation of NO/cGMP pathway (Zhang et al., 2007). Additionally, As-IV (orally, 40–80 mg kg d^−1^) improved the expression of eNOS, SOD and GSH-Px in the thoracic aorta and decreased ROS levels in a streptozotocin-induced diabetic rat model. It was suggested that As-IV ameliorates endothelial damages in the thoracic aorta of diabetic rats via regulating levels of ROS and calpain-1 (Nie et al., 2019).

#### Cornuside

Cornuside (Co, 100 μM) relaxed PHE (3 μM) pre-contracted rat aortas, which was inhibited by endothelial removal, L-NAME and ODQ rather than diltiazem (10 μM), TEA (100 μM), glibenclamide (10 μM), indomethacin (1 μM), atropine (1 μM) or propranolol (1 μM). Co also increased cGMP levels in HUVECs, which was inhibited by L-NAME (10 μM) or ODQ (1 μM). Therefore, Co displayed vasodilatory activity through activating the NO/cGMP pathway (Kang et al., 2007).

#### Chrysin Glucoside

Chrysin glucoside (Cg) could inhibit NE (1 μM)-induced contractions (IC_50_ 52 μM) in rat aortas, which was inhibited by L-NAME and endothelial removal. Cg (2.5 mg kg^−1^) significantly increased urine flow, glomerular filtration and electrolyte excretion (Na^+^/K^+^) in rats. Additionally, Cg (i.v. 10 mg kg^−1^) caused an immediate decrease in the mean arterial blood pressure of the anaesthetised rats, which was inhibited by L-NAME (Kang et al., 2007).

#### Tilianin

Agastache mexicana is used as an antihypertensive drug in Mexico. Tilianin (Ti, 0.002–933 μM), derived from A. mexicana, produced significant relaxation in aortic rings pre-contracted by NE (0.1 μM) or serotonin (5-HT, 100 μM). This relaxation was markedly abolished by endothelium removal, L-NAME (10 μM), TEA (5 mM), 4-AP (0.1 μM) or ODQ (1 μM) but not by indomethacin (10 μM) or atropine (1 μM). Additionally, Ti (50 mg kg^−1^, orally) significantly reduced systolic and diastolic blood pressure in an SHR (Kang et al., 2007). The vasodilatory activity of Ti was concerned with endothelial cells or K^+^ channels.

#### Jujuboside B

*Zizyphi Spinosi* Semen (ZSS) has protective effects against myocardial ischemic injury and hypertension. Jujuboside B (Ju), obtained from ZSS, reduced the tension of rat aorta with intact endothelium. However, the vasodilatory activity of Ju was attenuated by L-NAME, KN93, EGTA, SKF96365, iberiotoxin and glibenclamide rather than indometacin. Therefore, Ju displays its vasodilation through promoting eNOS levels and inhibiting K^+^/Ca^2+^ channels (Zhao et al., 2016).

#### Glucosyl hesperidin

Researchers have found that hesperidin possessed anti-oxidant, anti-hypertensive. Moreover, glucosyl hesperidin (Gh) has antihypertensive effects and improves lipid metabolism. Gh (50 mg kg d^−1^, orally) could reduce blood pressure in SHR and enhance endothelium-dependent vasodilation induced by acetylcholine. Additionally, Gh improved endothelial function by inhibiting the expression of nicotinamide adenine dinucleotide phosphate oxidase in SHR aorta (Yamamoto et al., 2008).

### Terpenoids

#### Ent-Trachyloban-14, 15-Dione

*Croton zambesicus* is used to treat hypertension in Benin. Ent-trachyloban-14, 15-dione (DT10), separated from *Croton zambesicus*, relieved K^+^-induced (100 mM) contractions in rat aortas (−logIC_50_ 6.3), which was significantly decreased by L-NAME (−logIC_50_ 5.7). Furthermore, DT10 inhibited K^+^-evoked contractions in aorta rings and SH-SY5Y (human neuroblastoma cells) (Martinsen et al., 2010).

#### Sesquiterpenoids

Zerumbone resisted aortas contracted by K^+^ (60 mM, IC_50_ 16 μM) (Fusi et al., 2015). Sesquiterpenoids [curcumanes A, B, C, and (±) D], which were isolated from Curcuma longa, also have significant vasodilation. They could significant resist KCl-induced aortic contractions in rats (EC_50_ 2.40, 0.91, 6.61, 14.56, and 16.03 μM, respectively) and curcumanes A, B, and C also inhibited PHE-induced aortic ring contractions in rats (EC_50_ 3.37, 0.83, and 4.26 μM, respectively). Additionally, curcumane C promoted the growth of human umbilical vein endothelial cells, which was inhibited by L-NAME. Curcumanes A and B produced vasodilation through regulation of VDCC and ROCC (Qiao et al., 2019: Liu et al., 2019). Therefore, sesquiterpenoids with significant activities should be paid more attention in the prevention and treatment of CCVDs.

### Coumarin

#### Imperatorin/Isoimperatorin

Imperatorin and isoimperatorin were shown to relax rat aorta contractions by PHE. The effect of imperatorin was significantly stronger than that of isoimperatorin. However, this activity was inhibited by endothelial removal and L-NAME (Nie et al., 2009).

#### (+)-Cis-4′-O-Acetyl-3′-O-Angeloylkhellactone

(+)-cis-4′-O-acetyl-3′-O-angeloylkhellactone (Al) relaxed rat aortas pre-contracted by PHE (1 μM, EC_50_ 17.8 μM) which was diminished by endothelial removal, L-NAME (100 μM) or methylene blue (30 μM). However, this function was not eliminated by indomethacin (30 μM), atropine (0.1 μM), triprolidine (10 μM), TEA (10 mM) and propranolol (3 μM). These results suggested that activity is mediated by Ca^2+^ channels and the NO/cGMP pathways (Lee et al., 2002).

### Others

#### Perillaldehyde

Perillaldehyde (Pe), is major compound from aqueous extract of Perilla leaves, improves NO levels in VSMCs. Pe (0.01–1 mM) also resisted aorta contraction by prostaglandin F-2*α* or NE, which was weakened by L-NAME, endothelial removal, propranolol, theophylline, TEA or glibenclamide. Therefore, the vasodilatory effect of Pe was concerned with blockage of Ca^2+^ channel (Takagi et al., 2005). Further, Pe (150 mg kg^−1^) reduced aortic atherosclerotic plaques, improved endothelial function, increased tetrahydrobiopterin and NO levels in carotid arteries of mice and rats fed high-fat diet (Yu and Liu 2018).

#### Cinnamaldehyde

Cinnamaldehyde (Ci), separated from Cinnamomi Cortex, has anti-platelet aggregation by regulating arachidonic acid. Ci (1–1,000 μM) also resisted rat aortas contracted by prostaglandin F-2α (5 μM), NE (0.1 μM) or K^+^ (60 mM). The activity was not inhibited by indomethacin, propranolol (10 μM), theophylline (100 mM, phosphodiesterase inhibitor), TEA (1 mM) or glibenclamide, instead of L-NAME (100 mM) (Yanaga et al., 2006). Further, Ci inhibited LTCC in mice ventricular myocytes and mesenteric artery SMCs (Buglak et at., 2018), (Alvarez-Collazo et al., 2014). The vasodilatory effect was related to endothelium cells and the Ca^2+^ channels.

#### Phthalides

*Ligusticum chuanxiong* is used for CCVDs to promote the circulation of blood and removed stasis in TCM. Ligustilide (Li), main phthalides of *L. chuanxiong*, was used to treat CCVDs. Li was shown to relax rat mesenteric arteries pre-treated by KCl, CaCl_2_, NA or 5-hydroxytryptamine (5-HT). But the activates was not affect by propranolol, glibenclamide, TEA and BaCl_2_. It was indicated that Li induces vasodilation through regulating VOCC and ROCC rather than endothelial cells (Cao et al., 2006). Further, the recent research found that the activity of phthalide dimers was generally superior to that of monomeric phthalides. Phthalides dimers, such as Chuanxiongdiolides R4 and R6, inhibited KCl-induced (60 mM) vasoconstriction. Moreover, Chuanxiongdiolides R4 and R6 also significantly inhibited the LTCC subunit *α*-1c (Cav1.2) (Tang et al., 2020).

#### Isosteviol

Isosteviol (Is, 10 μM) significantly relaxed the vasopressin (10^−8^ M)-/K^+^ (100 mM)-induced vasoconstriction in aortic rings, which was resisted by apamin and glibenclamide rather than K^+^ (30 mM). Therefore, this effect might be attributive to inhibition of KATP or SKCa (Wong et al., 2004).

#### Piceatannol

Piceatannol (Pi) caused relaxation in aortas pre-contracted by PHE (EC_50_ 2.4 μM). This effect was reduced by endothelial removal, L-NAME, methylene blue, ODQ, 4-AP and TEA rather than indomethacin, atropine, propranolol, nifedipine, BaCl_2_ or glibenclamide. Moreover, charybdotoxin and iberia toxin (BKCa channel blockers) could also reduce the activity of Pi. Therefore, this vasodilation of Pi may be related to the activation of BKCa and NO pathway (Oh et al., 2007b).

#### Eudesmin

Eudesmin (Eu) induced relaxation of rat aortic pre-contracted by PHE (IC_50_ 10.69 μg ml^−1^), which was blocked by endothelial removal, L-NAME, ODQ, indomethacin and diphenhydramine (type 1 histamine receptor), instead of atropine, propranolol and glibenclamide. This effect was mediated by regulation of histamine receptors and NO/prostaglandin pathways (Raimundo et al., 2009).

#### Brazilin

Studies have found that brazilin (Br) has anti-diabetic, anti-inflammatory, anti-asthmatic, anti-platelet aggregation, anti-tumour and anti-oxidant. Further, Br resisted the NE/K^+^-induced contraction of aortic rings (EC_50_ 83.51 and 79.79 μM, respectively), which was significantly attenuated by endothelium removal, L-NAME, methylene blue or indomethacin. Thus, the activity of Br was related to inhibition of ERK1/2 phosphorylation or blockage of Ca^2+^ channels (Yan et al., 2015).

#### Tanshinone IIA

Tanshinone IIA (Tan IIA) could reduce infarct area and blood pressure in hamsters. Moreover, Tan IIA caused endothelium-dependent relaxation, which was blocked by oestrogen receptor antagonist ICI 182 and 780. Therefore, the activity of Tan IIA was related to oestrogen receptor, NO channels and ERK1/2 phosphorylation (Fan et al., 2011).

#### Sodium Danshensu

Sodium danshensu (So) has anticoagulation and arrhythmia resistance on myocardial ischaemia-reperfusion injury. Moreover, So (1–3 g L^−1^) inhibited the PHE-/K^+^induced contraction of rat aortic, which was partially antagonised by TEA and apamin (SKCa blocker). However, the vasodilatory effect was not abolished by iberiotoxin (BKCa blocker), BaCl_2_ and glibencalmide (Zhang N. et al., 2010). Thus, the vasodilation of So was concerned with SKCa rather than BKCa, Kir and KATP.

#### Caracasanamide

Caracasanamide (i.v.) was shown to reduce blood pressure and increase cardiac muscle strength, respiratory rate and tidal volume in rats. Additionally, it also induced vasodilation in rats through acting on cardiac *β*1-adrenergic receptors (Delle Monache et al., 1993).

#### Others

Other lignans (saucerneol, saucerneol D and machilin D) exhibited vasodilation in rat aortic treated by PHE (10 μM, EC_50_ 2.2, 12.7 and 17.8 μM, respectively). The activities were significantly inhibited by L-NAME or endothelial removal. Additionally, saucerneol and sacerneol D were shown to significantly reduce left ventricular pressure (Oh et al., 2008).

### Clinical Application

**FIGURE 5:**
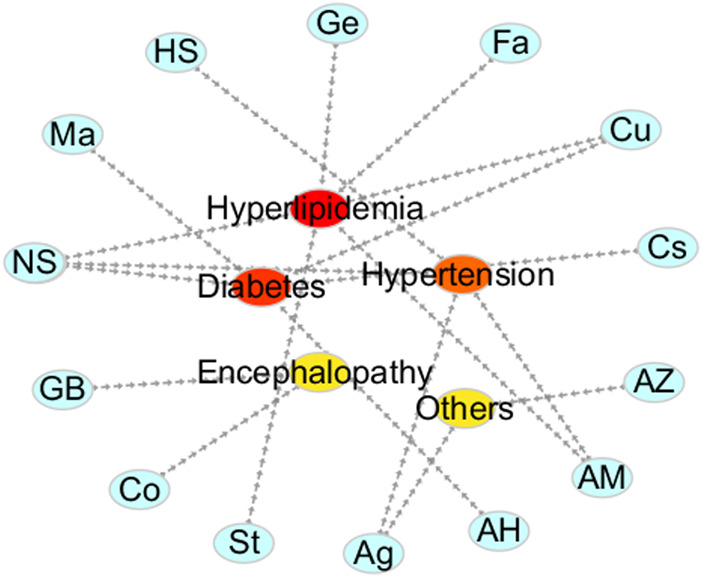
Clinical application of some TMPs and natural products. The vasodilating TMPs and natural products are mainly used in the treatment of diabetes, hypertension, hyperlipidemia and some encephalopathy in the clinic.

We also summarized the clinical applications of TMPs and natural products with vasodilatory activies (Figure 5). TMPs and natural products, listed in Figure 5, were mainly used in the treatment of diabetes, hypertension, hyperlipidaemia, and some encephalopathy, which demonstrated the relationship between the vasodilatory activity and the treatment of CCVDs. Some TMPs, such as *M. Vulgare and Nigella sativa*, etc, combined with conventional therapeutics improved the efficacy and tolerability of the drugs, and reduced their adverse reactions and side effects. Other TMPs, such as such as *Hibiscus sabdariffa* L, showed obviously antihypertensive effect. However, the detailed analysis of the drug’s underlying mechanism was not performed. In this present study, the role of TMPs and natural products in the prevention and treatment of CCVDs was positive and encouraging, but there were serious limitations in the druggability studies of this field due to lack of researches on toxicology and pharmacokinetics of TMPs and natural products with vasodilatory actives.

#### Diabetes

In addition to the medical treatment with glbenclamide, the patients suffering from Ⅱ diabetes were treated with *M. vulgare* (Ma, 3 weeks). The results showed that Ma could reduce levels of blood glucose (0.64%), cholesterol (4.16%) and TG (5.78%) in patients. *C. obtusifolia* lowered blood glucose (15.25%), cholesterol (14.62%) and TG (42.0%), respectively (Herrera-Arellano et al., 2004a). Therefore, it showed significant differences between the hypoglycemic effect produced by *C. obtusifolia* and *M. vulgare*. The type II diabetes treatment with curcumin (Cu, 1,000 mg d^−1^) plus piperine (absorption enhancer, 10 mg d^−1^) or placebo plus standard care for 12 weeks, significantly reduced the levels of TC (−21.86 vs.−17.06), non-HDL-C (−23.42 vs.−16.84) and lipoprotein (1.50 vs. −0.34), and increased the levels of HDL-C (1.56 vs.−0.22) as compared with the placebo group (Panahi et al., 2017). *Nigella sativa* (NS) (Kaatabi et al., 2015), *Artemisia herba alba asso* (AH) (Al-Waili 1986) and *Coriandrum sativum* (Cs) (Waheed et al., 2006) can significantly reduce fasting blood glucose in type Ⅱ diabetes. Furthermore, NS could significantly reduce fasting blood glucose, glycated hemoglobin and glutathione, but also improve the levels of total antioxidant capacity, SOD, catalase and glutathione.

#### Hypertension

NS oil (5 mL d^−1^, 8 weeks) significantly reduced diastolic and SBP in healthy volunteers without adverse effects. But there was no effect on levels of HAA, alanine aminotransferase, alkaline phosphatase, and creatinine (Fallah Huseini et al., 2013). *Hibiscus sabdariffa* L (HS, 9.6 mg d^−1^) also significantly reduced SBP in 30–80 year old hypertension patients, and there was no significant difference with captopril (50 mg d^−1^) (Herrera-Arellano et al., 2004b). Furthermore, aged garlic (Ag, 480/960 mg d^−1^, 12 weeks) significantly reduced SBP in hypertension patients (Ried et al., 2013).

#### Hyperlipidemia

NS (Sabzghabaee et al., 2012) (2 g d^−1^, 4 weeks) and Strawberries (Basu et al., 2014) (St, 5 or 50 g d^−1^, 12 weeks) significantly reduced serum malondialdehyde, TC, TG, LDL-C or HDL-C in adults. Ferulic acid (Fa, 1 g d^−1^, 6 weeks) could reduce levels of TC, TG, LDL-C or HDL-C in hyperlipidemia patients (Bumrungpert et al., 2018). AM (300 mg d^−1^, 2 months) and Cu (1 g d^−1^, 8 weeks) reduced levels of TC, LDL-C, HDL-C, TG and non- HDL-C in metabolic syndrome (MS) patients (Sikora et al., 2012). Additionally, Genistein (Ge, 54 mg d^−1^, 6 months) improved brachial artery vasodilation in postmenopausal women with MS, while lowering levels of TC, TG, and homocysteine (Irace et al., 2013). Furthermore, Cu (500 mg·d^-1^, 10 weeks) reduced body mass index, waist circumference, hip circumference and HDL-C levels in obese girls, but there was no significant difference with control groups (Panahi et al., 2014).

#### Encephalopathy

*Ginkgo biloba* extract (GB, 120 mg d^−1^, 52 weeks) improved cognitive performance in dementia patients and improved ADAS-Cog, GERR and CGIC scores in patients. In addition, GB leaves also improved the prognosis with acute ischemic stroke and increased the NIHSS score in patients. GB leaves (240 mg d^−1^, 3 months) could improve memory and attention in senile Alzheimer’s disease (Bars et al., 1997; Maurer et al., 1997; Oskouei et al., 2013). Coriander (Co, 4 weeks) also relieved migraine compared with control group by the Akaike criteria (Mansouri et al., 2015).

## Conclusion

This review discussed TMPs and natural products with vasodilation *in vitro*. Their possible mechanisms and clinical application were also summarised. Notably, TMPs with vasodilation are mainly from Compositae while natural products are flavonoids. The vasodilatory function of TMPs and natural products is mainly attributed to regulation of eNOS or Ca^2+^ channels. Further, we analysed briefly the structure-activity relationship of the compounds with significant vasodilatory effects. The vasodilation of the natural compounds was related to the number and position of double bonds, carbonyls, phenolic hydroxyl groups, and methoxy groups. Overall, these evidences suggested that TMPs and natural products are emerging alternatives for the prevention or treatment of CCVDs such as hypertension.

It was foreseeable that they will receive more attention in the future, although there were still some limitations based on literatures. Firstly, the current thoracic aortic models were usually used as the main mode to investigate the vasodilation, whereas different vessels, such as cerebral artery, abdominal aorta and mesenteric artery, have not sufficiently explored (Pfaltzgraff and Bader 2015). Secondly, the various channel blockers, such as TEA, BaCl_2,_ and 4-AP had been applied to block K^+^ channels. However, the relationship between the vasodilation of TMPs and natural products and other, such as KCNQ, TRPC, and TACC, had not been adequately investigated. Therefore, the underlying mechanisms, especially in relationship with various types of ion channels such as KCa, should be further explored. Thirdly, the clinical researches on the vasodilatory activity of TMPs and natural products is severely insufficient in this regard, although they exhibited remarkable potential in animal models. In addition, current the clinical studies on TMPs and natural products also have some deficiencies. For example, mechanisms of action usually remain unknown and only a small number of patients had been reported in almost all the related literatures. Finally, the application of TMPs is troublesome owing to difficulties in source identification, active ingredients, quality standard and mechanism study. Generally, natural products always maintain unsatisfactory druggability owing to their poor oral bioavailability, low plasma concentrations and so on. The ingested natural products was either excreted unabsorbably or metabolized rapidly after absorption, such as apigenin (Tang et al., 2017), sinomenine (Chen W. et al., 2009) and Kaempferol (Calderón-Montaño et al., 2011), etc.

However, developments in technologies such as metabolomics, proteomics and genomics will facilitate the application of TMPs and natural products in the treatment of CCVDs (Harvey et al., 2015). The bioavailability of natural products will be greatly improved by amelioration of hydrophilicity or alteration of administration modes. For instance, the curcumin nanoparticles with higher hydrophilicity achieved by loading into sophorolipid micelles had an appreciably higher bioavailability than that of free curcumin crystals (Peng et al., 2018). In addition, compared to oral administration, nasal administered paeonol was absorbed rapidly in rats (Adki and Kulkarni, 2020). The bioavailability of intravenous injection of cinnamaldehyde was also superior to that of oral administration (Zhu et al., 2017), etc.

In brief, we are still optimistic about the prospect of TMPs and natural products. TMPs will be used as alternative drugs and nutritional supplements, while natural products will be considered as potential therapies for CCVDs in the future. This study provides comprehensive and valuable references for the prevention and treatment of hypertension and CCVDs and sheds light on the further studies in this regard. In the next few years, it is necessary to investigate absorption, distribution, metabolism, and excretion of TMPs and natural products with vasodilation *in vivo*. Also, the activities of the major metabolites of these natural resources should be concerned.

## Abbreviations

SBP, systolic blood pressure; TC, total cholesterol; LDL-C, low-density lipoprotein cholesterol; HDL-C, high-density lipoprotein cholesterol; TG, triglycerides; non-HDL-C, non-high-density lipoprotein cholesterol; MS, metabolic syndrome; HAA, horizontal aspartate aminotransferase; ADAS-Cog, Alzheimer’s disease assessment scale-cognitive subscale; GERRI, geriatric evaluation by relative’s rating instrument; CGIC, clinical global impression of change; NIHSS, National Institutes of Health Stroke Scale.
