# Supplementary material for: Review of Natural Resources With Vasodilation: Traditional Medicinal Plants, Natural Products, and Their Mechanism and Clinical Efficacy
Source: Front Pharmacol. 2021 Apr 1;12:627458. doi: 10.3389/fphar.2021.627458 (PMC8048554; doi:10.3389/fphar.2021.627458)
Supplement: Supplementary file 1 [file table1.docx]

**Table S1 Vasodilatory mechanism of TMPs**

| **TMPs** | **Extraction solvent** | **ODQ** | **Pro** | **MB** | **Indo** | **Atr** | **L-NAME** | **En-de** | **Wor** | **Tha** | **Dil** | **Gli** | **Tea** | **BaCl_2_** | **4-AP** | **Re** |
| --- | --- | --- | --- | --- | --- | --- | --- | --- | --- | --- | --- | --- | --- | --- | --- | --- |
| **RC** | **Ehanol** | ***** | **#** |  | **#** | **#** | ***** | ***** | ***** | ***** | ***** | **#** | **#** |  |  | **Su et al. 2014** |
| **EB** | **Ehanol** |  |  |  |  |  |  | **#** |  |  |  | ***** | ***** | **#** | **#** | **Pan et al. 2008** |
| **VA** | **Ehanol** |  | ***** | ***** | ***** | ***** | ***** | ***** |  |  |  | ***** | ***** | ***** | ***** | **Taiwo et al. 2010** |
| **TP** | **Water** | **#** | ***** | ***** | ***** | ***** | ***** |  |  |  |  | **#** | **#** | ***** | **#** | **Salahdeen et al. 2015** |
| **AH** | **Ehanol** | ***** |  | ***** | **#** | **#** | ***** | ***** |  |  |  | **#** | **#** |  |  | **Skiker et al. 2010** |
| **GP** | **Water** |  |  |  | ***** |  | ***** |  |  |  |  |  |  |  |  | **Hoe et al. 2011** |
| **FCE** | **Ethyl acetate** |  | **#** | ***** |  |  | ***** | ***** |  |  |  | ***** | **#** | **#** | **#** | **Jiang et al. 2005** |
| **MH** | **Water** |  |  |  | ***** |  | ***** | ***** |  |  |  |  |  |  |  | **Brixius et al. 2006** |
| **WS** | **Methanol** |  |  |  | ***** |  | ***** | ***** |  |  |  |  |  |  |  | **Hernández-Pérez et al. 2014** |
| **AM** | **Water** |  |  |  |  |  | ***** |  | ***** |  |  |  |  |  |  | **Kim et al. 2013** |
| **SC** | **Methanol** | ***** | **#** | ***** | **#** | **#** | ***** | ***** |  |  |  | **#** | **#** |  |  | **Kang et al. 2005** |
| **CR** | **Ehanol** |  |  |  |  |  | ***** |  |  |  |  |  |  |  |  | **Khan et al. 2015** |
| **Co** | **Ehanol** |  |  |  |  |  |  |  |  |  |  |  | **#** | **#** |  | **Jabeen et al. 2009** |
| **GU** | **Ehanol** |  | ***** | ***** | ***** | ***** | ***** |  |  |  |  | ***** |  |  | ***** | **Tan et al. 2017** |
| **AI** | **Ehanol** |  |  |  | **#** | **#** | ***** | ***** |  |  |  |  |  |  |  | **Maciel, Dias and Medeiros 2004** |
| **SA** | **Total alkali** |  | **#** |  | **#** |  | **#** | **#** |  |  |  |  |  |  |  | **Zhang et al. 2009** |
| **MV** | **Essential oil** |  |  |  | ***** | **#** | ***** | ***** |  |  |  |  |  |  |  | **Guedes et al. 2004** |
| **ZC** | **Chloroform** |  |  |  |  |  |  | * |  |  |  | **#** |  |  | * | **Senejoux et al. 2010** |
| **SM** | **Water** |  |  | ***** |  |  | ***** | ***** |  |  |  |  |  |  |  | **Kamata et al. 1998** |
| **CF** | **Chloroform** | **#** | ***** | ***** | **#** |  | ***** |  |  |  |  | ***** | ***** | ***** | ***** | **Yam et al. 2016b** |
| **AM** | **Dichloromethane** |  |  |  |  |  |  |  |  |  |  |  | ***** |  |  | **Flores-Flores et al. 2016** |
| **WO** | **Ehanol** |  |  | **#** | **#** |  | **#** | **#** |  |  |  |  | ***** |  |  | **Naseri et al. 2008** |
| **AG** | **Ehanol** |  |  |  | ***** |  | ***** |  |  |  |  |  |  |  |  | **Takashima et al. 2017** |
| **AZ** | **Hydroalcoholic** | ***** |  |  | **#** |  | ***** |  |  |  |  | **#** |  |  | **#** | **Pinto et al. 2009** |
| **DO** | **Methanol** |  |  |  |  |  | ***** | ***** |  |  |  |  |  |  |  | **Liang et al. 2018** |
| **NS** | **Ehanol** |  |  |  | **#** |  | **#** |  |  |  | ***** | ***** | ***** |  |  | **Niazmand et al. 2014** |
| **GB** | **Ehanol** |  |  |  | ***** |  | ***** |  |  | ***** |  |  | ***** |  |  | **Kubota et al. 2001**  **Kubota et al. 2006**  **Tada et al. 2008**  **Nishida and Satoh 2004** |
| **SG** | **Hydroalcohol** |  |  |  |  |  |  | ***** |  | ***** |  |  |  |  |  | **Ayele, Urga and Engidawork 2010** |
| **JH** | **Hydroalcohol** |  |  |  |  |  |  |  |  |  |  | ***** | ***** |  | ***** | **Andrade et al. 2016** |
| **MBK** | **Ethanol** |  |  |  |  |  | ***** | ***** |  |  |  |  |  |  |  | **Oh et al. 2007a** |
| **HL** | **Water** |  |  |  | ***** |  | ***** |  |  |  |  |  |  |  |  | **Figard et al. 2008** |
| **SIL** | **Petroleum ether** |  | **#** | ***** | **#** | **#** | ***** | ***** |  |  |  | **#** |  |  |  | **Suresh Kumar, Patel and Saraf 2008** |
| **HS** | **Methanol** |  |  | ***** | **#** | ***** | ***** |  |  |  |  |  |  |  |  | **Ajay et al. 2007** |
| **JF** | **Ethanol** |  |  |  | **#** |  | ***** | ***** |  |  |  | **#** | ***** | ***** | ***** | **Yin et al. 2014** |
| **HSG** | **Ethanol** |  |  |  | ***** |  | ***** | ***** | ***** |  |  |  |  |  |  | **Ferreira et al. 2007** |
| **PP** | **Water** |  |  |  |  | **#** | **#** | ***** |  |  |  |  |  |  |  | **Khonsung et al. 2011** |
| **TS** | **Aqueous**  **Methanolic**  **Methylene chloride** |  |  |  |  |  | ***** | ***** |  |  |  |  |  |  |  | **Tom et al. 2010** |
| **GUB** | **Procyanidin** |  |  |  |  |  | ***** | ***** |  |  |  |  |  |  |  | **Magos et al. 2008** |
| **NSP** | **Ethanol** |  |  |  |  | ***** | ***** | ***** |  |  |  |  |  |  |  | **Senejoux et al. 2012** |
| **PAM** | **Water** |  |  |  |  |  | ***** |  |  |  |  |  |  |  |  | **Ojewole et al. 2007** |
| **SA** | **Water** |  | ***** |  |  |  |  |  |  |  |  | ***** | ***** |  |  | **Nguelefack et al. 2015** |
| **BK** | **Water** |  |  |  |  |  | ***** | ***** |  |  |  |  |  |  |  | **Ushida et al. 2008** |
| **CA** | **Ethanol** |  |  |  |  | ***** | ***** | ***** |  |  |  |  |  |  |  | **Shah and Gilani 2011** |
| **CL** | **Methanol** |  |  |  |  |  |  |  |  |  |  | ***** | ***** | ***** | ***** | **Adaramoye et al. 2009** |
| **Ru** | **Water** | ***** |  | ***** |  |  | ***** | ***** |  |  |  |  |  |  |  | **Kim et al. 2004b** |
| **GA** | **Ethanol** |  |  |  | ***** |  | ***** |  |  |  |  | ***** |  |  |  | **Chokri et al. 2012** |
| **MI** | **Ethanol** | ***** |  | ***** |  |  | ***** |  |  |  |  | ***** | ***** |  | ***** | **Crestani et al. 2009** |
| **EG** | **Water** |  |  | ***** |  |  | ***** |  |  |  |  |  |  |  |  | **Tibiriçá et al. 2007** |
| **GAE** | **Hexane** |  |  |  | **#** | ***** | **#** | ***** |  |  |  |  |  |  |  | **Wansi et al. 2012** |

**Table S1**: **The mechanism of vasodilation of some TMPs were described.** “*****”shows inhibitory activity; “#”shows no inhibitory activity. Pro: propranolol; MB: methylene blue, Indo: indomethacin; Atr: atropine; En-de: endothelium-dependent; Wor: wortmannin; Tha: thapsigargin; Dil: diltiazem; Gil: glibenclamide; Tea: tetraethylammonium; 4-AP: 4-aminopyridine; Re: reference.
